# Antenatal small-class education versus auditorium-based lectures to promote positive transitioning to parenthood – A randomised trial

**DOI:** 10.1371/journal.pone.0176819

**Published:** 2017-05-02

**Authors:** Vibeke Koushede, Carina Sjöberg Brixval, Lau Caspar Thygesen, Solveig Forberg Axelsen, Per Winkel, Jane Lindschou, Christian Gluud, Pernille Due

**Affiliations:** 1 The National Institute of Public Health, University of Southern Denmark, Copenhagen, Denmark; 2 The Danish Health Authority, Copenhagen, Denmark; 3 Copenhagen Trial Unit, Centre for Clinical Intervention Research, Copenhagen, Denmark; TNO, NETHERLANDS

## Abstract

Prospective parents widely use education to gain information about, e.g., labour and parenting skills. It is unknown if antenatal education in small classes is more beneficial for parenting stress and parenting alliance compared with other types of antenatal education. In the present randomised trial, we examined the effect of antenatal education in small classes versus auditorium-based lectures on perceived stress, parenting stress, and parenting alliance. A total of 1,766 pregnant women were randomised to receive: antenatal education in small classes three times in pregnancy and one time after delivery, each session lasted 2.5 hours, versus standard care consisting of two times two hours auditorium-based lectures. Previous analysis of the primary outcome showed no difference between intervention and control group. Here we conduct an exploratory analysis of three secondary outcomes. Effects of the interventions on parents’ global feelings of stress at 37 weeks gestation and nine weeks and six months postpartum and parenting stress nine weeks and six months postpartum were examined using linear regression analyses and mixed models with repeated measurements. The effect on parenting alliance six months postpartum was examined using the non-parametric Wilcoxon rank-sum test. Antenatal education in small classes had a small beneficial main effect on global feelings of stress six months postpartum and a statistically significant interaction between time and group favoring antenatal education in small classes. The P values of intervention effects on parenting stress and parenting alliance were all larger than the threshold value (0.05).

## Introduction

Antenatal education aims to help prospective parents prepare for childbirth and parenthood. The education uses a range of educational and supportive measures to help parents understand social, emotional, psychological, and physical needs during pregnancy, labour, and parenthood [1].

### Changes of antenatal education over time

In most Western countries antenatal education is well-established, but the form and content have changed markedly over time. Both antenatal education in small classes with group discussions and lectures in large auditoriums have been used. These educations have been offered without evidence of an effect of specific types of antenatal education on relevant outcomes [1]. Over the past years, Danish antenatal education has gradually moved away from large-scale auditorium-based lectures to antenatal birth and parent preparation classes in small groups for all expectant parents. However, there is a lack of evidence from trials favouring antenatal education in small classes over auditorium-based lectures.

The main focus of most antenatal education has been on birth and breastfeeding; while information on parent-child attachment and psychosocial aspects that relate to couple- and parenthood has generally not been covered [2]. The Danish Health Authority emphasises that several studies describe parents’ wishes to discuss aspects related to the social, emotional, and psychological aspects of parenthood, and how to interact with their newborn baby, in addition to gaining information about the delivery, and recommend that antenatal education comprises these aspects [3]. Antenatal education in small classes is more costly than auditorium-based lectures which may be an incentive for the hospitals to offer the auditorium-based approach.

In 2012, when the present trial was initiated, antenatal education was still offered as auditorium-based lectures in the Capital Region of Denmark. This Region was therefore an ideal setting to conduct a randomised trial of antenatal education in small classes versus auditorium-based lectures.

### Antenatal education in small classes

In recent time, principles of adult learning have been given more weight in antenatal education and all health-promotion has been recommended to provide opportunities for people to learn skills in order to practice desired behaviours [4]. Further, it is highlighted that people learn more effectively in a group setting, where they have the opportunity to assume different roles, to observe others’ perspectives, to interact regularly, and to supplement one another [5].

Qualitative research on pregnant women’s preferences in relation to antenatal education has suggested that women prefer antenatal education in small classes leaving possibilities for discussion, suggestions for practicing skills, and encouragement for participants to get to know and support one another [6].

### Stress and parenting alliance

Becoming a parent is a challenging experience for most people. Parenting stress, defined as the aversive psychological reaction to the demands of being a parent [7], and global feelings of stress, i.e., having difficulties coping with everyday life, is a common concern faced by many parents [8]. How parents experience and adapt to the transition to parenthood and how it affects the couple relationship depends on the resources available to them [8, 9]. Important resources for a positive transition and for parenting are social support [10–12], confidence in one’s ability to cope with demands [13–16], and parenting alliance [17–19].

Because of its influence on parenting and potentially detrimental effects on the wellbeing of children, parents, and the family as a whole, numerous studies have examined the correlates or consequences of parenting stress [9, 20–23] Yet studies on the prevention of parenting stress and global feelings of stress are scarce.

Certain parenting programmes have shown to be effective in reducing stress and improving the short-term psychosocial wellbeing of parents [24]. The majority of parenting programmes are delivered after the child has reached a minimum of three years as part of secondary, high-risk approaches to prevention [24, 25]. It has been argued on theoretical grounds and from a public-health point of view that these programmes would be more effective if delivered as part of a population approach, i.e., delivered to all parents preventing problems before they arise and promoting child and parent health and wellbeing [26–29]. Many prospective parents look to antenatal education to gain information about labour, infant and postnatal care, and parenting skills [1]. Antenatal education may therefore offer a suitable and non-stigmatising setting for promoting parenting alliance, social support, and confidence in ability to cope with the demands of parenthood, thereby reducing parenting stress and promoting wellbeing.

Results from the few existing randomised trials on antenatal education lend support to the notion that structured antenatal education focusing on promoting social support, coping strategies, and parenting alliance could be an effective population-based approach to reducing parenting stress among newborn parents [19, 26, 30]. However, the trials are all based on a small number of participants. Due to the scarce evidence from randomised trials, there is a need to conduct high-quality, randomised trials with adequate sample sizes [1, 31–33].

We, therefore, conducted a randomised trial to examine the effect of an antenatal education programme including psychosocial aspects of parenting in small classes versus standard education carried out as auditorium-based lectures on birth and breastfeeding–the NEWBORN trial [34].

The aim of this article is to present the effects of the experimental intervention in the NEWBORN trial on the secondary outcomes: maternal global feelings of stress, parenting stress, and parenting alliance. The primary outcome of the trial; use of epidural analgesia–as a proxy for coping during labour–has been reported elsewhere [35]. We found no statistically significant difference between the intervention and control groups regarding the primary outcome. In line with the statistical analysis plan published in the trial protocol, the results of the analyses of the secondary outcomes reported in this article should therefore be interpreted as explorative [34]. The purpose of this article is therefore to examine the effect of the NEWBORN programme as inspiration for the planning and selection of outcomes in future trials.

## Methods

### Study design and participants

We used data from the randomised trial: NEWBORN–preparation for birth and parenting. The trial is registered at ClinicalTrials.gov (ID: NCT01672437). A detailed description of the trial is published in the design article [34] and the trial protocol which can be accessed here: http://www.interventionsforskning.dk/userfiles/files/nyfdt/projektprotokol-version-23_2.pdf [36]. The NEWBORN trial took place at the largest birth site in Denmark, Hvidovre Hospital, situated in the Copenhagen Capital Region. More than 6,000 deliveries take place at Hvidovre Hospital every year and the catchment area comprises a diverse population regarding socio-demographic characteristics.

Women were enrolled in the trial from 10+0 to 20+0 weeks of gestation. Inclusion criteria were expectant women, ≥18 years old at enrolment, singleton pregnancy, due to give birth at Hvidovre Hospital, having the ability to speak and understand Danish, and signing the informed consent form.

Pregnant women were recruited from August 2012 to May 2014. The women received a written invitation to participate in the trial prior to their first visit to the midwife along with an informed consent form. Invitations were followed up by a phone call from a project employee. Initially, only primiparous women were eligible for participation, but due to slow recruitment also multiparous women were included approximately six months into the recruitment period in order to ensure adequate statistical power [36].

#### Randomisation

Baseline data were collected using a web-based questionnaire prior to randomisation (approximately in gestation week 18). A project employee performed individual web-based randomisation according to a computer-generated allocation sequence of 1:1 with varying block sizes developed by The Copenhagen Trial Unit and concealed to the investigators. The allocation was stratified for parity (primiparous or multiparous) and vulnerability (yes or no as evaluated by their general practitioner at the first pregnancy consultation in gestation week 6–10). Vulnerability was based on eight criteria set forward by the Danish health authorities, for example; former or current psychiatric disorder, adverse psycho-social background, or concerns about parenting skills. The general practitioner categorised the woman as vulnerable if she met one or more of these criteria. For non-vulnerable women the randomisation block sizes were 10 and 20, for vulnerable women block sizes were 4 and 6. These block sizes were used for primiparous as well as multiparous women and were unknown to the investigators. All citizens in Denmark have a unique personal identification (CPR) number and the randomisation programme was set up to confirm the existence of the CPR number.

#### Experimental intervention group

The experimental intervention was developed using a systematic framework for health promotion programme planners; Intervention Mapping [37]. This systematic framework aids effective decision making at each step in intervention planning, implementation, and evaluation.

Women in the intervention group received an antenatal education programme aimed at strengthening relationships and improving information and problem solving skills for expectant parents in order to ease birth and the transition to parenthood. The programme was designed based on the recommendations for antenatal care from the Danish Health Authority [3].

The programme included short verbal presentations from the group facilitator, individual exercises, short film presentations, and time for discussions and reflection. Parents were given homework in the form of minor exercises in preparation to each session. Educational subjects were: the transition to parenthood; couple communication; birth; breastfeeding; and taking care of a newborn. A patient-network website was created as a supplement to the sessions. The programme was focused on psychosocial aspects and parenting resources important to the birth process, parenting and mental health, that appear amenable to change, i.e.: social support, parenting alliance, cognitive coping, and parenting skills:

1. Social support: formal and informal, emotional, informational and instrumental. Groups of 6–8 couples were offered three times 2.5 hour sessions during pregnancy (gestation week 25, 33, and 35) and one session five weeks after expected due date. The groups were composited to enable participants establish relations with other expectant parents in their local area. Sessions were led by a midwife and the postnatal session included a health visitor. A patient-network website enabled parents to gain further information, communicate with other parents and consult online with a midwife and a health visitor.
2. Parenting alliance: the programme had a component supporting the couples in the transition to parenthood and couple communication.
3. Cognitive coping: sources of self-efficacy were embedded into programme content and delivery, an environment enabling parents to discuss feelings and concerns was aimed for, enhancing their awareness of own resources, problem-solving strategies, and future challenges in parenting and emotional regulation
4. Parenting skills: increasing information and exercises with feedback, e.g., on recognising signs and symptoms of thriving in the newborn, couple communication etc.

The approach aimed at strengthening relationships and improving information and problem solving skills for expectant parents in order to ease birth and the transition to parenthood.

To maximise the potential for population uptake classes were established at three local midwifery sites. A comprehensive guide and education material for course facilitators was developed, and facilitators, i.e., midwives and health visitors were trained at one-day workshops. The framework for the classes was based on an estimate of adequate time allocated to each subject, and what service providers deemed a sustainable service.

More details of the programme has been presented elsewhere [34]. A total of 25 midwives and six health visitors with varying professional seniority and teaching experience facilitated the 110 classes. Facilitators joined the trial voluntarily and were not selected by the trial group. The facilitators followed a detailed teaching manual developed for the trial [38].

#### Control group

Women in the control group were offered the standard education from Hvidovre Hospital at the time consisting of two antenatal lectures of two hours each concerning delivery and breastfeeding in an auditorium with participation of up to 250 people.

To avoid contamination of conditions midwives teaching the small class education programme were not allowed toteach the antenatal lectures in the control group.

Participants in the intervention group as well as the control group were permitted to make use of concomitant birth and parent education.

### Blinding

It was not possible to blind participants or service providers. Data were blinded by a data manager, and the investigators were blinded to participants’ intervention category during data assessment and analyses. Participants’ intervention category was not revealed to the investigators until the Steering Committee of the trial had drawn two conclusions about intervention effects on outcomes under code [39, 40].

### Ethics

The Danish National Committee of Health Research Ethics reviewed the study protocol of the NEWBORN trial and concluded that formal ethical approval was not required. This decision is registered under the Capital Region’s ethics committee (protocol number H-4-2012-FSP) and the trial is further registered and listed in the Danish Data Protection Agency (reference number 2011-54-1289). The study complies with the Helsinki Declaration. Oral and written consent was obtained from all participants.

### Times of assessments

Data stems from web-based questionnaires sent to the women at 37 weeks gestation (FU1), 9 weeks postpartum (FU2), and 6 months postpartum (FU3). Baseline data were collected at approximately 18 weeks gestation.

### Outcomes

Global feelings of stress were measured by the *Perceived Stress Scale (PSS)* [41] at baseline, FU1, FU2 and FU3. The scale consists of 10 items. All answers are added together to a sum score with a potential range from 0–40 with a low score being favourable.

Parenting stress was measured by the *Swedish Parenthood Stress Questionnaire (SPSQ)* [42] at FU2 and FU3. This scale consists of 34 items. All answers are added together to a sum score with a potential range from 34–170 with a high score being favourable.

Parenting alliance was measured by the *Parenting Alliance Measure (PAM)* [43] at FU3. All answers are added together to a sum score with a potential range from 19–95 with a high score being favourable. The original PAM scale consists of 20 items. For the NEWBORN trial one item regarding punishment of the child was excluded due to it not being appropriate as the questionnaire was distributed when the child was six months old.

### Baseline data

The following variables were used for examination of baseline differences: Educational level was measured by the question: “What is your highest completed education?” The educational level was dichotomised into ≤medium tertiary education versus higher tertiary education. Body Mass Index (kg/m^2^) was calculated using information on pre-pregnancy weight and height reported by the woman at the first pregnancy consultation at the general practitioner. Living with child’s father was self-reported by ticking the response category “Living with the child’s father” in the question: “Which grown-ups do you live with?” Planned pregnancy was self-reported by the question: “Is this pregnancy planned, partly planned or not planned” and dichotomised into: planned (yes or partly) versus not planned. Self-rated physical and mental health was measured by the items: “How would you describe your physical/mental health status altogether?” Response categories: “Excellent; very good; good; poor; very poor”. Self-rated physical/mental health was dichotomised into excellent; very good versus good; poor; very poor. Feeling of stress was measured by the item: “Do you feel stressed?” Response categories: “no; yes, a little; yes, moderately; yes, a lot”. Stress was dichotomised into no versus yes, a little; yes, moderately; yes, a lot. Antenatal depressive symptomatology was measured by the Edinburgh Postnatal Depression Scale [44]. Women with a score of 13 or more were categorised with antenatal depressive symptomatology.

### Adherence to the interventions

Data on adherence to the interventions was collected by tablets after each session. Due to technical breakdowns, data on adherence was also collected by questionnaires at FU1 and FU2. The measurement used in the analyses is a combined variable consisting of information obtained from tablets and supplemented with questionnaire data from FU1. We defined adherence to the experimental intervention as participation in all three sessions before birth and using the website at least once. We defined adherence to the control intervention as participation in both lectures before birth. If information from the FU1 questionnaire could not be obtained, data from FU2 was used.

Data on use of concomitant birth and parent education was collected by questionnaires at FU1 and FU2.

### Adverse outcome

As a potential adverse outcome we examined antenatal and postnatal depressive symptomatology among participants measured by the Edinburg Postnatal Depression Scale [44] at FU1 and FU2.

### Sample size and power calculations

The sample size was based on the primary outcome of the trial, use of epidural analgesia [36, 45]. The usage of epidural analgesia has previously been published [35] and is not the focus of the current paper.

Given the sample size of 1756 participants, we assessed the corresponding power for each of the three secondary outcomes which we report here. This was done in order to assess the validity of any statistical result. The power estimations showed that we were able to detect a minimal relevant difference of 1 point on PSS using a standard deviation (SD) of 6 points [46], 0.1 point on SPSQ using a SD of 0.5 points [42], and 4 points on PAM using a SD of 20 points [43] with a power of 0.94 or more [36].

## Statistical analyses

This paper presents results from analysis of the secondary outcomes: global feelings of stress, parenting stress, and parenting alliance. Data were analysed according to the intention-to-treat principle and following the recommendations of the CONSORT statement [47, 48].

We found no statistically significant difference between the intervention and control group regarding the primary outcome [35]. To account to multiplicity, we did, as planned in our protocol, accept the null hypotheses of the remaining outcomes without test [36]. For hypothesis-generating purposes we now analysed the data according to the following principles.

**Fig 1:**
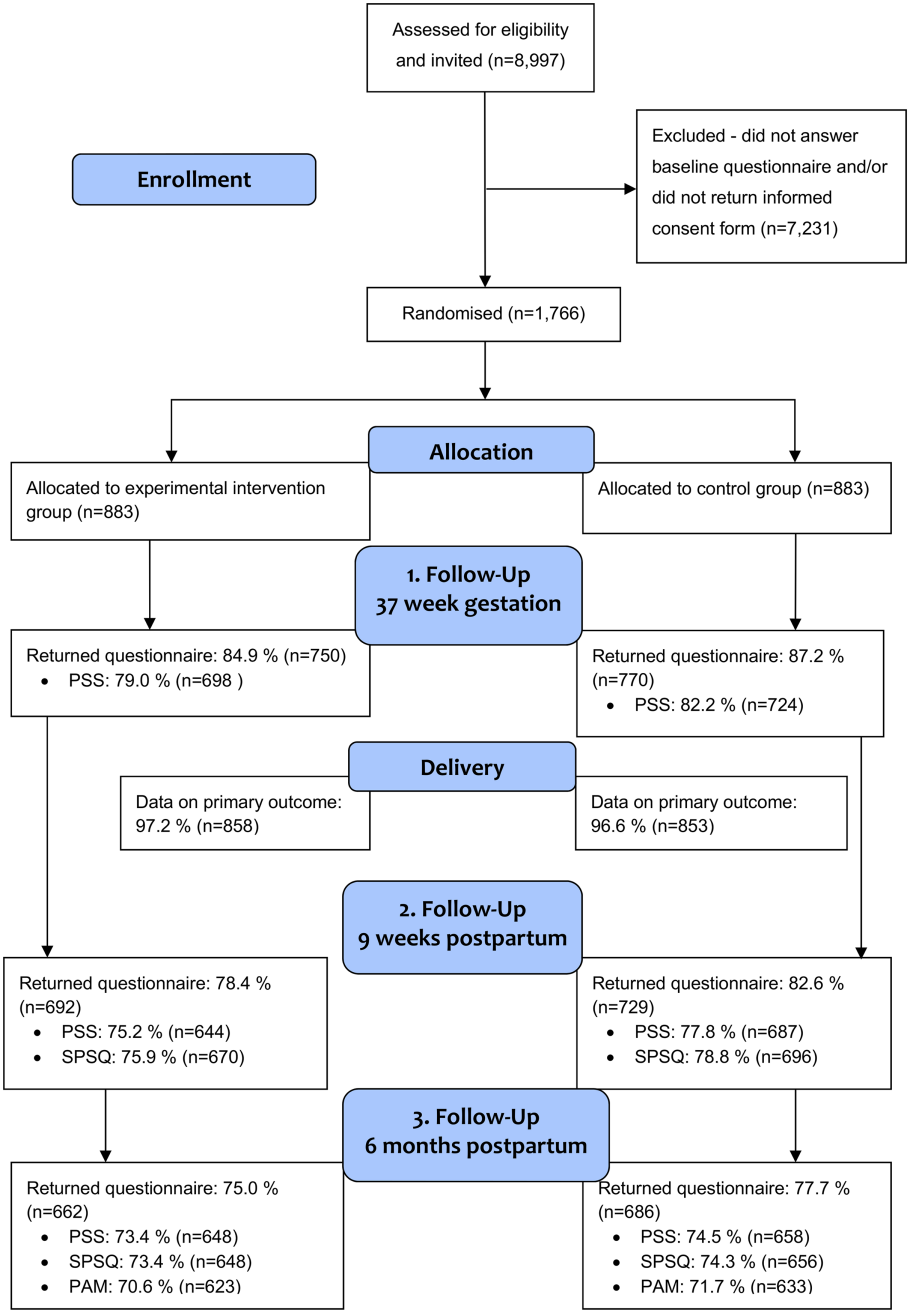
Flow diagram of recruitment, randomisation, and response in the NEWBORN trial.

Differences between intervention and control group were tested as follows:

1. We examined differences in mean values (MD) with 95% confidence intervals (95% CI) between the two groups at FU1, FU2, and FU3 (PSS) and FU2 and FU3 (SPSQ) using a general linear model. We analysed changes of the mean scores over time between groups of PSS and SPSQ using a mixed model with repeated measurements for continuous outcome measures. Analyses were performed with the unstructured covariance structure. For PSS, the fixed effect included a linear and a quadratic time component and the corresponding interactions with group. The quadratic time component was added to the model to increase model fit. The interaction term between time and group was used to evaluate the changes in PSS over time and to compare these changes between intervention and control group. For SPSQ, the fixed effect included a linear time component and group, but no interaction term due to lack of a baseline measurement for this outcome. Due to item missingness in the SPSQ scale it was chosen prior to data analysis to use the average score for each individual instead of the total sum score. The overall proportions of reporting for each of the three outcomes are presented for all time points in the trial flow diagram (Fig 1). There was no pattern in missingness on separate items and missingness was likely due to electronic questionnaire layout.
  Analyses were adjusted for the trial stratification variables; parity and vulnerability. In the analyses of PSS, additional adjustment for the baseline PSS value was performed. Due to non-normally distributed residuals, analyses were performed with square root transformation of PSS.
2. The PAM scale had a negatively skewed distribution and analyses of difference between groups at FU3 were performed using non-parametric analysis with Wilcoxon rank-sum test.

### Missing data

Missing values on PSS and SPSQ were accounted for by using multiple imputation technique. Twenty imputed datasets were created using 13 baseline variables measuring, e.g., relationship satisfaction, stress, and socio-demographic factors for prediction of missing values. Also, intervention group, parity, vulnerability as well as the outcome measures at each measurement time point were included for prediction. To account for missing values on PAM, conditional mean imputation was performed. Based on the same variables used to predict missing values in the multiple imputation procedure missing values on PAM was assigned a mean value conditioned on the level of the prediction variables. The primary analyses were based on the result of the analyses with imputed data.

Differences in proportion of the adverse outcomes; antenatal and postnatal depressive symptomatology between groups at FU1 and FU2 were tested by χ^2^-test.

### Additional analyses

We conducted a *post-hoc* analysis with the aim of examining the impact of concomitant birth and parent preparation. We excluded participants who made use of concomitant birth and parent education in both intervention and control group.

The compliance with the randomised interventions was not 100%. We therefore planned per protocol analyses in our trial protocol. Definition of per protocol conditions was done prior to data analysis following the definition used for the primary outcome [35]. The per protocol populations were defined as follows:

1. Participants in the intervention group who participated in all three sessions before birth and used the website at least once versus all participants in the control group.
2. Participants in the intervention group who participated in all three sessions before birth and used the website at least once versus participants in the control group who participated in both antenatal lectures.

These additional analyses were performed on the imputed dataset using a general linear model. We analysed the impact of adherence to the intervention as mean differences (95% CI) of PSS (FU1, FU2, and FU3) and SPSQ (FU2 and FU3). Impact of adherence on the effect of the intervention on PAM was performed using non-parametric analysis with Wilcoxon rank-sum test.

The following differ from the original analysis plan [34]: we analysed the effect of the intervention over time in a mixed model with repeated measurements instead of the pre-planned analyses of area under the curve. Decisions on changes were made prior to data inspection.

All statistical analyses were performed using SAS v. 9.3, SAS Institute Inc. The level of significance was set to 0.05.

## Results

### Participant flow, baseline data, and adherence

During the recruitment period, 8,997 women were invited to participate in the NEWBORN trial. Of these, 1,766 women (19.6%) accepted participation and were randomised– 883 women to the intervention group versus 883 to the control group. At baseline, the characteristics among the intervention and control groups were well balanced (Table 1).

**Table 1:** Baseline characteristics of women enrolled in the NEWBORN trial (n = 1,766).

	Experimental intervention (n = 883)	Control intervention (n = 883)
Age at delivery in years (mean (SD))*	30.7 (4.1)	30.8 (4.1)
Nulliparous % (n)	89.1 (787)	88.9 (785)
Vulnerable women % (n)**	4.8 (42)	4.8 (42)
Educational level (medium/long) % (n)	75.6 (659)	76.5 (663)
Body Mass Index kg/m^2^ (mean (SD))*	23.4 (4.0)	23.3 (4.1)
Living with child’s father (yes) % (n)	93.8 (828)	96.0 (848)
Planned pregnancy (yes/partly) % (n)	90.9 (801)	91.5 (808)
Self-rated physical health status (excellent/very good) % (n)	68.6 (605)	71.2 (628)
Self-rated mental health status (excellent/very good) % (n)	72.0 (635)	75.9 (669)
Not feeling stressed % (n)	48.2 (425)	49.2 (433)
Edinburgh Postnatal Depression Scale score of 13 or more % (n)	4.8 (42)	3.2 (28)

* Based on women with birth data (n = 1,711).

** Vulnerability evaluated by the general practitioner at the first pregnancy consultation in gestation week 6–10.

The proportion of women returning questionnaires at the three follow-up time points were 86.1% (FU1), 80.5% (FU2), and 76.3% (FU3) (Fig 1). The response proportions were slightly higher in the control group compared with the intervention group.

### Effect of the intervention

#### Main analyses

Perceived Stress Scale: We found no statistically significant difference in mean values of square root PSS between the two groups at FU1 (MD: -0.06, 95% CI: -0.14 to 0.02, p = 0.13) or FU2 (MD: -0.06, 95% CI: -0.15 to 0.04, p = 0.23) in the linear regression analyses. At FU3, the mean square root PSS adjusted for baseline PSS was slightly, but statistically significantly lower among participants in the intervention group compared with the control group (MD: -0.10 (95% CI: -0.20 to -0.01), p = 0.04) (Table 2). Using the non-transformed PSS scale, the mean difference is 0.47 points out of the 40 points one can achieve on the PSS scale (Table 2).

**Table 2:** Mean differences (95% CI) of PSS and SPSQ between intervention and control group at the three follow-up time points.

	Experimental intervention group Mean	Control intervention group Mean	Mean difference (95% CI)	p-value	Mean difference (95% CI)*	p-value*	Mean difference (95% CI)**	p-value**
PSS***								
FU1	3.22 (10.18)	3.25 (10.50)	-0.03 (-0.12–0.07)	0.56	-0.03 (-0.12–0.07)	0.57	-0.06 (-0.14–0.02)	0.13
FU2	3.24 (10.53)	3.27 (10.72)	-0.03 (-0.13–0.08)	0.58	-0.03 (-0.13–0.07)	0.58	-0.06 (-0.15–0.04)	0.23
FU3	3.19 (10.19)	3.26 (10.66)	-0.07 (-0.18–0.03)	0.18	-0.07 (-0.18–0.03)	0.18	**-0.10** **(-0.20–0.01)**	**0.04**
**SPSQ**								
FU2	3.56	3.53	0.03 (-0.02–0.08)	0.27	0.03 (-0.02–0.08)	0.27		
FU3	3.55	3.53	0.02 (-0.03–0.07)	0.34	0.02 (-0.03–0.07)	0.34		

* Test for difference in means adjusted for parity and vulnerability.

** Test for difference in means PSS adjusted for parity, vulnerability and baseline PSS

*** PSS square rooted. Means represent means from the square-rooted transformations. Numbers in parentheses are calculated means.

**Fig 2:**
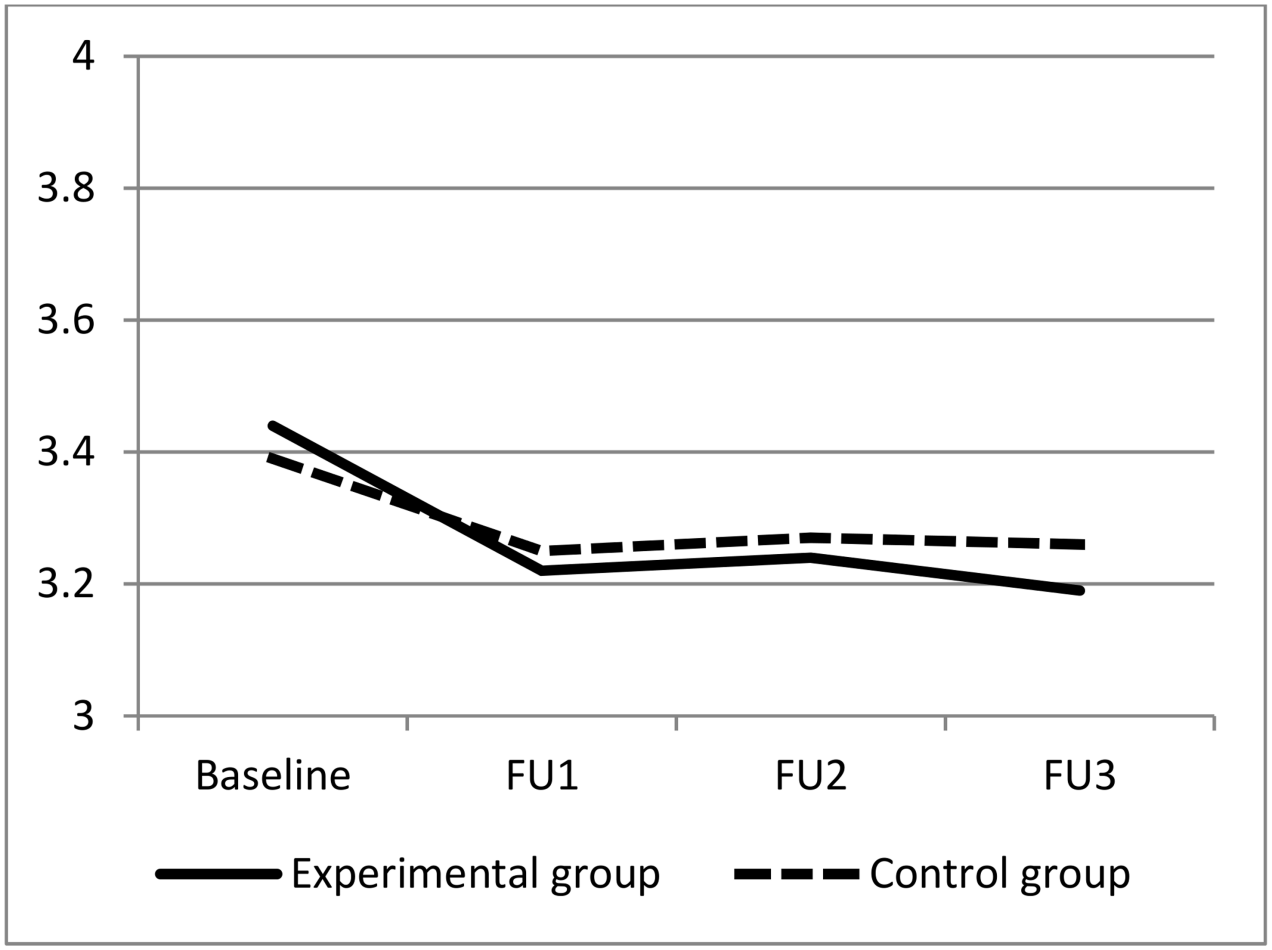
Comparison of square rooted mean PSS over time between intervention and control groups.

Fig 2 shows a graphical presentation of the mean square rooted PSS values for the two groups at baseline, FU1, FU2, and FU3. The intervention group had a slightly higher mean square root PSS at baseline but improved relative to the control group over time.

Results from the mixed-model analysis showed a small but statistically significant difference in change in mean square root PSS scores over time (p = 0.02).

**Fig 3:**
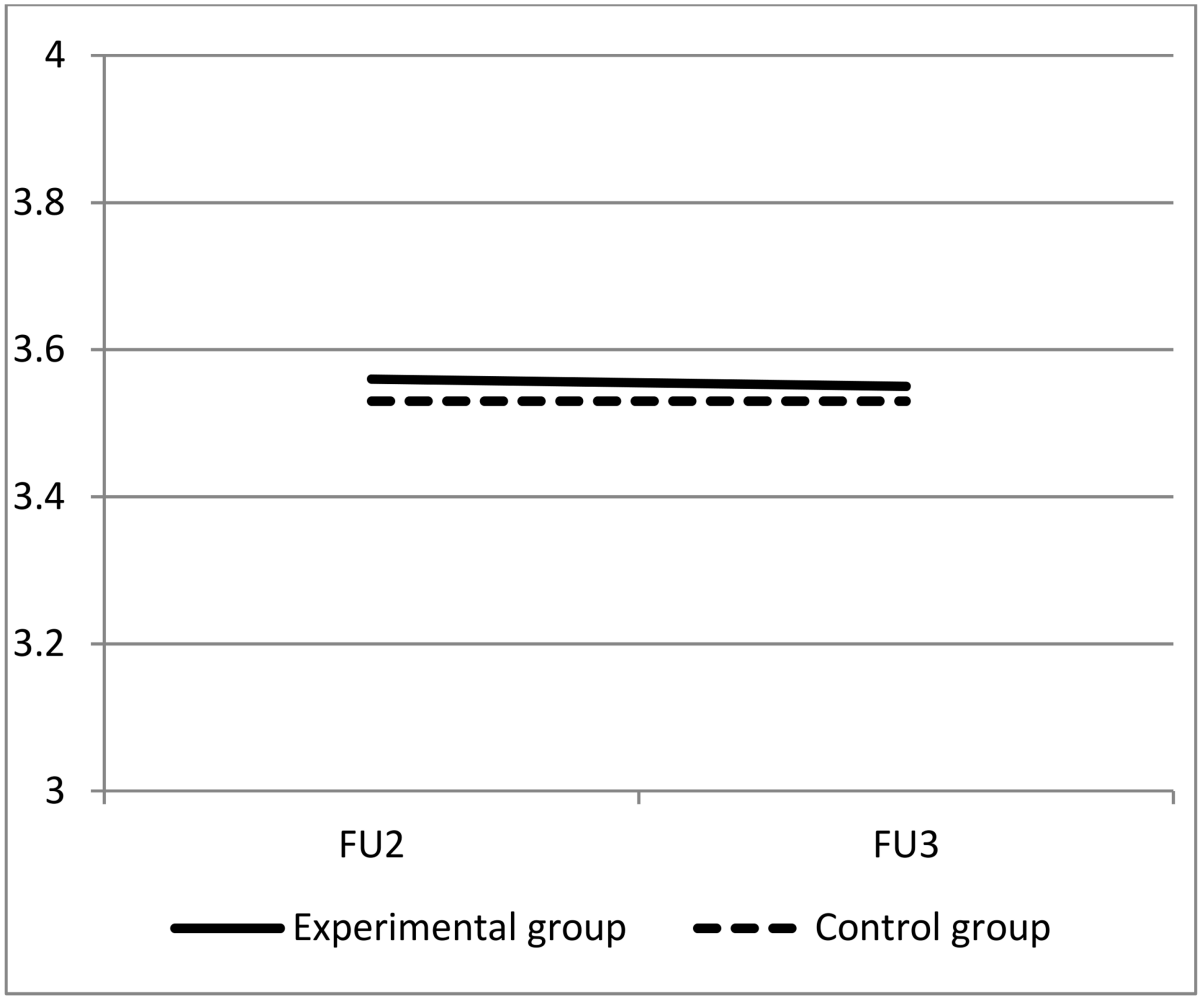
Comparison of mean SPSQ over time between intervention and control groups.

Swedish Parenthood Stress Scale: Linear regression analyses of SPSQ showed no statistically significant differences between the two groups at either FU2 (MD: 0.03, 95% CI: -0.02 to 0.08, p = 0.27) or FU3 (MD: 0.02, 95% CI: -0.03 to 0.07, p = 0.34) (Table 2). Fig 3 shows a graphical presentation of the SPSQ mean values for the two groups at FU2 and FU3.

Also, no statistically significant difference in mean SPSQ at the two time points were observed between groups in the mixed model analysis (p = 0.24).

Parenting Alliance Measure: The median value of PAM was 88 (interquartile range 79 to 93) in the intervention group and 87 (interquartile range 79 to 83) in the control group. The Wilcoxon signed-rank test showed no significant difference in median PAM score between the two groups at FU3 (p = 0.33).

When performing the same analyses using complete case data, results were consistent with the analyses performed on the imputed data for all three outcomes (results not shown).

Adverse outcome: There were no statistically significant differences between intervention and control group regarding antenatal and postnatal depressive symptomatology. At FU1, 5.6% of the women in the intervention group and 6.8% of the women in the control group were categorised with antenatal depressive symptomatology (p = 0.34). Also, at FU2 the difference in proportion with postnatal depressive symptomatology was not statistically significantly different between intervention group (5.8%) and control group (7.4%) (p = 0.24).

#### Additional analyses

Exclusion of women who made use of concomitant preparation education: A total of 25.0% of the participants in the intervention group and 38.7% in the control group attended concomitant antenatal preparation. We performed sensitivity analyses examining the effect of the intervention excluding women who made use of concomitant preparation education using linear regression models (PSS and SPSQ) and Wilcoxon rank sum test (PAM). Results were similar to the results from the intention-to-treat analyses with no substantial difference in the mean (PSS and SPSQ) or median values (PAM).

Per protocol analyses: A total of 68% adhered to the intervention, i.e., participated in all three sessions before birth and used the website at least once. In the control group, 59% of the participants attended both lectures. In the per protocol analyses where we examined the effect of the intervention among participants adhering to the intervention versus the entire control group, results were consistent with the intention-to-treat analyses. Also, in the per protocol analyses comparing the participants adhering to the intervention versus the control group participating in both antenatal lectures, results were consistent with intention-to-treat analyses.

## Discussion

Our hypothesis was that antenatal education classes in small groups could potentially promote parenting alliance, social support, and confidence in ability to cope with the demands of parenthood, thereby reducing maternal global feelings of stress, parenting stress, and promoting parenting alliance. The paper presents the analysis of secondary outcomes; hence we are unable to make any confirmatory claims concerning our findings.

We found no statistically significant effects of the NEWBORN programme on parenting stress measured nine weeks and six months after birth or parenting alliance measured six months after birth. For global feelings of stress, there was a statistically significant interaction between time and group favoring the intervention group. This difference between randomised groups appears to be driven by a statistically significant difference in perceived stress six months after birth and is limited to a modest difference between groups substantially lower than the predefined minimal relevant difference of 1 point [34]. Accordingly, this finding should be considered without clinical meaning.

To date only a few randomised trials on antenatal education have been conducted. Of them even fewer have examined the effect of antenatal education provided in small classes on stress or factors related to parenting alliance, and findings are inconsistent. An American trial by Schultz et al. found no effects of a couple-focused intervention consisting of an extra 24 weekly sessions compared with standard care on marital satisfaction among mothers and fathers six months and 5.5 years postnatally. Nor did they find any differences in relation to divorce or separation 5.5 years postnatally [49]. Results from another American trial by Feinberg et al. indicated significant effects of an eight session psycho-social prevention programme for couples, compared with a brochure on child care delivered to participants in the control condition, on co-parenting six months after delivery [19]. In a trial conducted in France and Spain, Ortiz Collado et al. found no effect of an antenatal psychosomatic programme designed to decrease depression among women at high risk of postnatal depression compared with standard care, consisting of eight large-group sessions on childbirth and pregnancy health, on dissatisfaction with the relationship 5–12 weeks after delivery [50]. A trial by Daley-McCoy et al. conducted in UK found that women who participated in an extra 2 hour session about relationship functioning experienced significantly less deterioration in relationship quality and couple communication compared with women who did not participate in this extra session (both intervention group and control group received antenatal education in small classes) [26]. In an Australian trial by Petch et al. they found that high-risk women receiving a couple relationship- and coparenting-focused education programme delivered through a 6 hour workshop and two home-visits reported higher relationship satisfaction, and were less intrusive in their parenting than high-risk women receiving a mother-focused parenting programme primarily delivered as telephone consultations [51]. None of these trials are directly comparable to our NEWBORN trial. Two of the trials were aimed at women at high risk of depression and relationship problems. In four out of the five trials the dose of the intervention, i.e., the amount of sessions offered was higher than in the NEWBORN trial. Finally, the content and delivery of the interventions as well as control conditions differed to the NEWBORN trial.

The NEWBORN programme was developed in line with recommendations set forward by the Danish Health Authority, i.e., all expectant parents should be offered antenatal birth and parent preparation classes in small groups with the opportunity to discuss feelings and concerns related to birth and parenthood [3]. The programme was developed using a systematic framework for health promotion program planners [37]. This systematic framework aids effective decision making at each step in intervention planning, implementation, and evaluation [37].

### Strengths and limitations

To our knowledge, the NEWBORN trial is the largest randomised trial on small-class antenatal education to date. We minimised the risk of bias in all important domains [52]. Although it was impossible to blind participants and investigators, we were able to blind all other aspects of the trial [35]. The analyses of the effect of the intervention were performed using imputed data taking into account the potential differential drop-out. This implies that we were able to conduct intention-to-treat analyses implying that the results are not biased by incomplete outcome data or selection bias.

We only have a follow-up period lasting till six months after due date. This leaves limitations regarding the assessment of participant-relevant outcomes, such as the child’s thriving as it grows up, the number of families that experiences divorces and break-ups, and child’s use of the health-care system in both the short and the long run. We assess these outcomes in the NEWBORN trial, but we range them in the outcome hierarchy as ‘exploratory’. This is done, as 1) we have very limited knowledge of the potential effect of antenatal education on these outcomes, and we have therefore not been able to perform power estimations, and 2) due to logistical and financial constraints. If additional funding can be obtained, data on all individuals can be sought in the national registers and long-term follow-up will be possible.

The trial recruited participants from a single hospital in Denmark, which may reduce the external validity of our findings. However, the intervention was delivered by 25 different midwifes and 8 different health-visitors in 3 different local sites, which in turn increases the generalisability. Furthermore, the trial had very wide eligibility criteria, leaving potential findings applicable to the entire Danish population. Although we used wide inclusion criteria, only a small proportion (11%) of the participants were multiparous.

The NEWBORN programme was delivered using a health promoting population-based approach. This approach has been suggested to have the advantage of reaching groups who may otherwise be difficult to reach. In the current trial, parents who participated had a higher education level compared to the general population of Copenhagen women in the same age group. This phenomenon is common in trials [53]. There was a high proportion of women with a university degree in our trial. The control group received standard care, i.e., auditorium-based lectures. This way of acquiring knowledge is common for women having attended university. Their ability to benefit from this type of information may have reduced outcome differences between the intervention group and the control group, while a more socially diverse group of participants in the trial might have increased the benefit of small class education. It is also possible that the lack of effect was due to a ceiling-effect as this particular group of women may already have a high level of communication skills and be used to working out differences and collaborating with their partners in a productive way, e.g., in relation to parenting alliance.

The educational discrepancies between the trial population and the background population may limit the generalisability of the trial results. It would be beneficial to conduct research focusing on the effect of the programme among subgroups, e.g., vulnerable women. Also, further in depth analyses taking implementation of the programme into consideration would contribute with more thorough knowledge of the impact of the programme.

The programme was developed with guidance from politicians and service providers to ensure that the programme would be feasible to implement in an everyday clinical practice setting if proven effective. It is possible that provision of a more comprehensive programme could lead to larger effects; however, in the current setting with limited healthcare resources, it is questionable if an expensive programme would be implemented.

We focused on conducting a trial using standard care as control condition. This is advantageous as the effect of the intervention is measured against the existing offer making decisions on change in provision of care more straightforward. Finally, the study population was recruited among a diverse population group and not limited to a high-risk population. This increases the likelihood of results be generalisable to a general population, but the low and socially skewed participation challenged this generalisability.

Results from the NEWBORN trial constitute a much-needed base for future trials and for decision-makers regarding the form and content of antenatal education.

### Implications for future research

In this paper, as in most studies on the effects of antenatal education, we have focused only on the mother’s transition into motherhood. Data from the partners were also gathered, and it would be relevant in future studies to explore the effect of the NEWBORN programme among the partners. This could bring valuable new insight to an area with limited knowledge.

## Supporting information

S1 Protocol (PDF)

S1 Checklist (PDF)
