# Supplementary material for: Antenatal small-class education versus auditorium-based lectures to promote positive transitioning to parenthood – A randomised trial
Source: PLoS One. 2017 May 2;12(5):e0176819. doi: 10.1371/journal.pone.0176819 (PMC5413036; doi:10.1371/journal.pone.0176819)
Supplement: S1 Protocol — (PDF) [file pone.0176819.s001.pdf]

Project **New**born –**P**reparation for **B**irth and **P**arenthood

PROTOCOL IDENTIFYING NUMBERS version 23

PROTOCOL VERSION DATE: 1/04 -2014

PROTOCOL REGISTRATION NUMBERS (CTU number: Nyfødt –DP -269, ClinicalTrials.gov identifier: NCT01672437, The Capital Region's ethics committee number: CVR/SE-nr: 30113713, protocol nr. H-4-2012-FSP, Data Protection Agency: CVR-nr. 11-88-37-29 J.nr. 2011-54-1289.)

Center for Intervention Research

Table of Content:

Wordlist and abbreviations 4

Abstract 5

1. Introduction and background 11

1.1 The condition and population 11

1.2 Investigational intervention 11

1.3 Antenatal education 11

1.4 Clinical data 13

1.5 The theoretical and conceptual framework: 13

1.6 Regimen rationale 16

1.7 Risk/benefits 17

1.8 Ethical consideration 17

2. Trial objectives and purpose 18

3. Trial design 19

3.1 Trial design 19

3.2 Randomisation 19

3.3 Trial intervention 19

3.4 Blinding 20

3.5 Duration 20

3.6 Discontinuation of individual participants 20

3.7 Intervention accountability 20

Center for Intervention Research

3.8 Data collection 21

4. Selection and withdrawal of participants 21

4.1 Inclusion criteria 21

4.2 Exclusion criteria 21

4.3 Participant withdrawal 21

5. Experimental interventions 21

5.1 Concomitant medication/treatment 26

5.2 Monitoring for participant compliance 26

6. Assessment of outcome measures 26

6.1 Efficacy variables 26

6.2 Method and timing 34

7. Statistical plan and data analysis 37

7.1. Sample size and power estimations 37

7.2 Statistical methods 38

7.3 Significance 39

8. Direct access to source data/documentation 39

9. Data handling and record keeping 39

10. Quality control and quality assurance 40

11. Trial organisation 40

12. Legal aspects 41

12.1 Finance and insurance 41

12.2 Publication plan 41

12.3 Spin-off projects 41

Center for Intervention Research

12.4 Intellectual property rights ... 42

13. Supplements ... 42

14. References ... 45

Wordlist and abbreviations

AND Antenatal depression

EPDS Edinburgh Postnatal Depression Scale

Gestation: The gestation period is the time in which a foetus develops, beginning with fertilization and ending at birth

MDI Major Depression Index (MDI)

NIPH National Institute of Public Health

Nulliparous : Never having given birth to a child

PND Postnatal depression

PP post-partum i.e. after birth

PSE Parenting self-efficacy

PSS Perceived stress scale

SOC Sense of coherence

Center for Intervention Research

Abstract

Background: The transition to parenthood brings about major changes in men and women's roles, responsibilities, and identities. Parents' well-being is fragile during this transition period, as many mothers and fathers, even those considered to be "low risk", experience it as a stressful time (1). How parents experience the transition and adapt to parenthood depends on the resources available to them (1, 2).

Parents are increasingly dependent on drawing on their own resources rather than on professional help and advice in the time following birth (3). Many parents attend antenatal classes to prepare themselves for birth and parenthood. Antenatal education has, however, been sensitive to opinions and trends and has undergone dramatic changes without us knowing much about its effects on relevant outcomes (4). Today antenatal classes are mainly held as lectures in auditoriums with focus on birth and breastfeeding. The Danish regions currently aim to implement antenatal birth and parent preparation classes in small groups for all expectant parents. However, it is unknown if 1) antenatal preparation in small groups is superior to standard care; 2) what elements the preparation should encompass to meet the needs of expectant parents today; and 3) what the cost-effectiveness of antenatal preparation in small groups is compared to large-scale preparation in auditoriums.

Objectives: The main objective is to compare parenting resources, health and thriving, and use of healthcares services in newborn families enrolled in a structured antenatal parenting program –The Newborn, preparation for birth and parenthood program - with those allocated to standard care.

We have developed a comprehensive research based parenting program in collaboration with midwives, health care visitors, psychiatrists, psychologists and family therapists, parents, and leading national and international researchers in this field.

We also aim to ensure careful implementation of the program, and to conduct a thorough process evaluation highlighting enabling factors and barriers to the implementation.

Finally we will conduct cost-effectiveness analysis using questionnaires and national registers of health care utilisation.

Design: Individually randomised trial sited at Hvidovre Hospital, a large birth clinic in the Copenhagen Capital Region.

Participants: 2350 prgenant women ≥ 18 years old, recruited before 20+0 weeks gestation, due to give birth at Hvidovre Hospital. Being legally able and willing to provide signed consent, and being fluent in Danish.

Intervention: Women are randomised to receive:

Center for Intervention Research

1) A research-based birth and parenting program. The intervention consists of 4 sessions in small groups that last for 2,5 hours per session at 25, 33 and 35 weeks of gestation, and a post-natal session 5 weeks after expected due date.

2) Standard care (control group). The pregnant woman and her partner are offered two antenatal lectures in an auditorium.

The allocation of participants to the intervention will be 1:1 to the intervention and the control group.

Methods: Data will be collected via questionnaires at baseline, 37 weeks gestation, 9 weeks post-partum, 6 months post-partum and 1 year post-partum, via the hospital obstetric database, and via the national registers. Analyses will be intention to treat as well as per protocol analyses – sensitivity analyses comparing results from the two will be conducted. Subgroup analysis will be conducted in relation to personal and demographic characteristics.

Process evaluation will be conducted using questionnaires and qualitative interviews. The incremental societal cost of the intervention will be computed and compared to the measured outcomes in a cost-effectiveness analysis with epidural use during labour as the primary outcome. Direct health care costs and productivity cost in terms of labour market participation and short term absence will be calculated.

Outcome measures:

Primary outcome: Epidural use during labour.

Secondary outcomes: Parenting stress, parenting alliance

Explorative outcomes: Obstetric intervention, antenatal and postnatal depressive symptomatology and anxiety, breastfeeding, use of healthcare services, smoking and medicine use, satisfaction with relationship, divorce/ family break-ups.

Intermediate outcomes: trust in own ability to cope with: 1) birth, 2) discharge 3) parenting, 4) breastfeeding; couple communication; social network and support

Center for Intervention Research

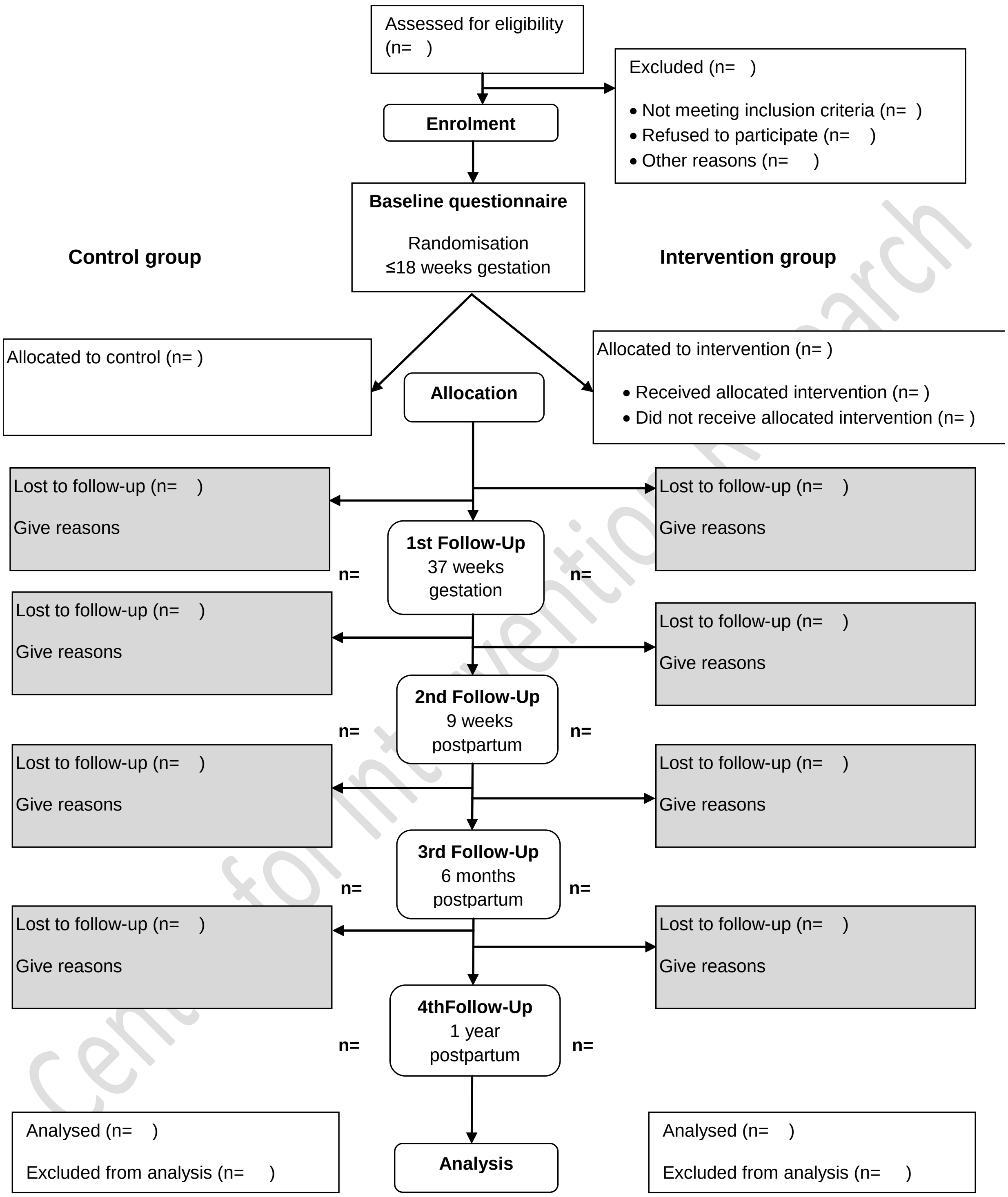

Figure 1: Flow-chart for the NEWBORN trial

Name and address of the sponsor of the trial:

Pernille Due, The National Institute of Public Health and Centre for Intervention Research, Øster Farimagsgade 5A 2nd floor, 1353 Copenhagen, 6550 77 77

Morten Grønbæk, Director of The National Institute of Public Health, Øster Farimagsgade 5A 2nd floor, 1353 Copenhagen, 6550 77 77

Coordinator and principal investigators responsible for the trial and institutions involved in the trial:

Co-ordinating and principal investigator: Vibeke Koushede,
Carina Brixval, Research Assistant
Solveig Forberg Axelsen, Research Assistant
The National Institute of Public Health, Øster Farimagsgade 5A 2nd floor, 1353 Copenhagen, 6550 7864

Marianne Skovby Chief midwife
Birthe Kjærgaard Vice-chief midwife
Randi Iversen Vice-chief midwife
Kirsten Marcussen, Co-coordinating senior midwife
Tom Weber, Chief of obstetrics, consultant obstetrician and gynecologist
Hvidovre Hospital
Gynækologisk/Obstetrisk Afdeling
Kettegård Allé 30
2650 Hvidovre

Christian Gluud MD, Dr. Med.Sci, Head of Department at The Copenhagen Trial unit
Jane Lindschou Hansen, MSc, Research associate
Per Winkel, Senior statistician
Janus Engstrøm, Data manager
The Copenhagen Trial unit
Rigshospitalet, Dept. 33.44
2100 Copenhagen Ø

Working group:

Anette Due Madsen, Psychologist and family therapist, Head of Centre for Family Development
Janne Ekstrøm Larsen, Midwife, MVO, IBCLC Hvidovre hospital
Karen Mylin, Health visitor
Nynne Ek Sindberg, midwife and lecturer at the Metropolitan School of Midwifery Education

Background group and Scientific support:

Anne Mette Skovgaard, Chief Child Psychiatrist and Research Leader at the Child and Youth Psychiatric Centre Glostrup, and Lecturer at the University of Copenhagen.
Naja Hulvej Rod, Head of the Copenhagen Stress Research Centre, Associate Professor, University of Copenhagen

Poul Videbæk, Professor and Executive Consultant at the Centre of Psychiatric Research, Aarhus University Hospital

Center for Intervention Research

Bodil Kirstine Møller Midwife, MTLD, Head of the school of Midwifery Education
Charlotte Nielsen, Chief Health visitor and member of the Capital Region Birth Planning Committee
Conny Kuhlman, Midwife at Hillerød Hospital
Laust Hvas Mortensen, Researcher, MSc in Public Health, PhD, University of Copenhagen
Mia Rasmussen, Member of Parenting and Childbirth (parenting NGO)
Rikke Damkjær Maimburg, Researcher midwife, MPH, PhD, University of Aarhus
Søren Brønchenburg, Member of Parenting and Childbirth (parenting NGO)
Tea Trillingsgaard, Mpsych(clin) ph.d.-fellow, University of Aarhus

International scientific support:
Gerjo Kok, Professor of Applied Psychology, Maastricht University, The Netherlands
John Lynch, Professor of Public Health at the University of Adelaide, Australia

Further collaboration:
Jesper Gravgaard, Head of Development, Rambøll Management Consulting, Aarhus
Thomas Hagen Holten (Jørgensen), Manager, MSc (Political Science), Rambøll Management Consulting, Aarhus
Jacob Frølund, Consultant, Rambøll Management Consulting, Aarhus

Facilitators - midwives:
Amina Aharaz
Anna Holm Bjerreskov
Anne Børglum Ensig
Anne Randløv
Cecilie Lawaetz
Charlotte Falk
Henriette Mørkholm
Janne Ekstrøm Larsen
Katja Richter
Line Kreibke
Lise Lotte Bisgaard
Louise Gravgaard
Louise Munk Haugaard
Maria Kongsgaard
Marie Hellstrøm
Marie Sommer
Mie Møller Hansen
Mille Bech
Mira Fromberg Gorlén
Pernille Lindgren
Rikke Bodenhoff
Trine Rosa
Facilitators - healthcare visitors:
Dorthe Eskling-Hansen
Eva Toftkjær Lisse

Center for Intervention Research

Ingrid Schäfer
Netta Gadiel
Nina Rafael
Sille Klyver
Vibeke Rose-Møller

Center for Intervention Research

Center for Intervention Research

1. Introduction and background

The following protocol is based on the Copenhagen Trial Unit's Standard Operating Procedures.

1.1 The condition and population

Every year approximately 60.000 children are born in Denmark. A body of research suggests that early parenting is key to child emotional and behavioral functioning (5). How parents experience the transition and adapt to parenthood depends on the resources available to them (1, 2). The transition to parenthood brings about major changes in men and women's roles, responsibilities, and identities. Parents' well-being is fragile during this transition period, as many mothers and fathers, even those considered to be "low risk", experience it as a stressful time (1), and a substantial number of new parents have difficulty navigating this period of change. Increased parenting stress during the first year of the infant´s life has been found to be an important risk factor for later behavioural problems in some studies (6, 7).

1.2 Investigational intervention

Two types of antenatal education will be compared:

Intervention: Project Newborn – preparation for birth and parenthood. Antenatal course in small groups consisting of four elements of 2.5. hour sessions each.

Control: Standard care, 2 antenatal lectures in the birth site auditorium

1.3 Antenatal education

A smooth transition into parenthood, and low stress in the parenting role is viewed by most psychologists and psychiatrists as critically important to positive parent and child outcomes (8). Becoming a parent is a challenging experience for most people and a substantial number of new parents have difficulty navigating this period of change (1, 2). Even those considered to be "low risk", experience it as a stressful time (1). Parents in many western countries often feel overwhelmed during the first year of infancy (9). Creating a healthy and secure family environment while adapting to the new role as parents, requires knowledge, support and resources (10). Central to how parents adapt to parenthood is the parent's perceptions of having access to available resources for meeting the demands of parenthood (2, 11).

In recent years antenatal and postnatal services have undergone marked changes (3) without us knowing much about the consequences of this. Parents are increasingly dependent on their ability to draw on their own resources rather than on professional help and guidance during pregnancy and shortly after birth.

Prospective parents often look to antenatal education to provide important information on issues such as decision making about and during labour, infant and postnatal care, breastfeeding and parenting skills (4). Antenatal education aims to help prospective parents prepare for childbirth and parenthood,

using a range of educational and supportive measures to help parents understand their own social, emotional, psychological, and physical needs during pregnancy, labour and parenthood (4). However, antenatal education has been sensitive to opinions and trends and has undergone dramatic changes without us knowing much about its effects on relevant outcomes, and evidence of the potential benefit of general antenatal education for childbirth or parenthood or both remains largely unknown (4).

Being prepared for childbirth and parenthood may help parents cope with many concomitant situations, and pregnant women are very amenable (by and large) to receiving and acting upon public health messages (12). Pregnancy is therefore by many considered an ideal period for health promoting and preventive interventions (12, 13).

The National Board of Health recommends that all expectant parents should be offered antenatal birth and parent preparation classes in small groups with the opportunity to discuss feelings and concerns related to birth and parenthood (14). They declare that little is known about what these classes should encompass in order to meet the needs of parents today, but that several studies describe parents wishes to discuss aspects related to the social, emotional and psychological side of parenthood, and how to interact with their newborn baby, in addition to gaining information about the delivery (14).

Qualitative studies among parents of young children suggest that small parent groups can provide parents with knowledge about family life, strengthen their social support networks, enhance their awareness of their own resources, and increase their confidence concerning their ability to cope (15). It is possible that antenatal education in small groups may similarly contribute to valuable social networks, enhance parents' awareness of their own resources, and increase their confidence in their ability to cope – with the delivery and with the parenting role.

Over the past years, Danish antenatal education has gradually moved away from large-scale auditorium-based education to antenatal birth and parent preparation classes in small groups for all expectant parents. However, it is unknown if 1) antenatal preparation in small groups is superior to standard care; 2) what elements the preparation should encompass to meet the needs of expectant parents today; and 3) what the cost-effectiveness of antenatal preparation in small groups is compared to large-scale preparation in auditoriums.

Antenatal classes are still offered as lectures in hospital auditoriums with a minimum of interaction with the audience in the Capital Region of Denmark, although this region is planning to implement birth and parent preparation in small groups. The Capital Region of Denmark is therefore an ideal setting for a randomised trial of group-based versus auditorium-based antenatal education.

In a healthcare system with limited resources it is important to develop an up-to-date antenatal preparation program and examine its efficiency and cost-effectiveness prior to implementation, in order to prevent excess obstetric procedures and use of health care, to improve newborn families' health and thriving, and to meet the aim of evidence based medicine and most value for money.

Center for Intervention Research

1.4 Clinical data

A Cochrane review from 2007 concludes that the effects of general antenatal education for childbirth or parenthood, or both, remain largely unknown (4). The review was edited in 2011 adding references for studies awaiting assessment (4). Primo 2012 we reviewed the updated references and searched the Cochrane Pregnancy and Childbirth Group's Trials Register by contacting the Trials Search Coordinator. The selection criteria for included studies were: randomized controlled trials of any structured educational program provided during pregnancy by an educator to either parent that included information related to pregnancy, birth or parenthood. The educational interventions could have been provided on an individual or group basis. We assessed trial quality for each of the included studies. Details of the randomization procedure, allocation concealment, and/or participant accrual or loss to follow-up for many of the trials were not reported. No consistent results were found. Hence results from the updated studies did not alter the overall conclusion that the effects of general antenatal education for childbirth or parenthood remain largely unknown. Previous studies have been difficult to interpret, mainly because of limitations in study design and small sample sizes, and many program evaluations do not utilize randomly assigned control or comparison groups. Cochrane reviews on randomised parent training programs suggest that parenting programmes have a potential role to play in the promotion of mental health (5, 16); however, they also highlight important limitations of the studies conducted e.g. incomplete outcome data, failure to report attrition, lack of long term follow-up etc. Finally, most current evidence from controlled trials address the use of parenting programs as part of secondary, high-risk approaches to prevention, although it has been argued that they would be more effective if delivered as part of a population-based approach (5, 17), in which they are offered to all parents with the aim of preventing problems before they occur, and promoting child and parent health.

1.5 The theoretical and conceptual framework:

The aim of the trial is to evaluate if antenatal birth and parent preparation in small groups can increase parenting resources thereby easing birth and creating a smoother and less stressful transition to parenthood. This in turn is hypothesized to improve health and thriving among newborn families and affect their use of healthcare services.

Subjective appraisal and self-reported measures

Parental belief systems and subjective appraisals of situations related to parenting as measured by self-report have both direct and indirect influences on parenting behavior and child outcomes. The indirect path is mediated by the dyadic interaction - thus the parent engages in interactions with the child based on his or her belief system, and child outcomes follow from these interactions. The direct path relates to the environment the parent creates or involves the child in and to the inferences the child makes about the parent's belief system (expectations) (2). This direct influence cannot be understood by observing a particular behavior, but can be determined only over a long history of parent-child interactions in a large variety of contexts. Thus behavioral observations are an inefficient and somewhat ineffective method of

Center for Intervention Research

assessing some of the direct influences of the impact of parental belief systems on their children. Direct assessment of parental belief systems by self-report has been suggested to be a more effective and practical methodology (2).

Definition of stress:

Perceived stress:

In this trial stress is examined using a psychological perspective which focuses on individuals' subjective appraisal of their abilities to cope with demands.

Some of the most important theorists in the field of psychological stress and coping are Lazarus and Folkman. They suggested that stress can be thought of as resulting from an imbalance between demands and resources or as occurring when pressure exceeds one's perceived ability to cope (18). Stress is considered a transaction between the person and the environment that is appraised as potentially dangerous to one's wellbeing (known as the Transactional Model). Two critical processes mediate this person-environment relationship: 1) The cognitive appraisal, which is an evaluative process that determines why and to what extent a particular transaction between the individual and the environment is stressful; and 2) Coping, the process through which the individual manages the demands of the person-environment relationship by identifying various resources - i.e. what can I do to handle this problem? The individual identifies coping strategies and manages emotions generated from the situation (18).

The model breaks the stressor-stress link by proposing that if stressors are perceived as positive or challenging rather than a threat, and if the stressed person is confident that he/she possesses adequate rather than deficient resources and coping strategies, stress may not necessarily follow the presence of a potential stressor (18). In other words, no event or situation in itself is inherently stressful; instead, the stressor is defined by the subjective judgment of the situation that is appraised as threatening, harmful, or taxing of available resources.

Parenting stress:

In 1992 the noted American psychologist with expertise in parenting behaviour Richard R Abidin presented a model on parenting stress and behavior (2) based on previous theoretical models (e.g. Belsky's model of determinants of parenting (19)) and on empirical findings on the role and functioning of parents. The dynamics of Abidin's model of parenting behavior (2) are quite similar to those suggested by Lazarus and Folkman (18) in their transactional model of stress and represents an explication of a specific application of Lazrarus and Folkman's general theory. Parenting stress is viewed as the result of a series of appraisals made by each parent in the context of his or her level of commitment to the parenting role.

According to Abidin parenting stress is the motivational arousal that encourages parents to utilize the resources available to them to support their parenting (i.e. what Lazarus and Folkman refer to as coping, the process through which the individual manages the demands of the person-environment

Center for Intervention Research

relationship by identifying various resources). The richness or paucity of resources available plays a key role in the ultimate parenting behavior (2). Based on empirical findings social network and support, parenting alliance, parenting skills and competencies, material resources, and cognitive coping are considered key resources for the role and functioning of parents (2).

In this trial we focus on participants' perceived levels of stress. Perceived stress is a measure of subjective appraisal of stress that takes the meaning and manageability of the event to the individual into account. This is important as individual characteristics (i.e. beliefs, values and commitments) and contextual factors (e.g. available social network and support) may conjointly influence the degree to which any particular event, in this case the transition to parenthood, may be stressful.

Definition of coping

There are various schools of thought within coping research. One fundamental question within this field concerns the choice of coping strategy, i.e. whether coping reflects a personal trait or style or rather a situation specific solution. Does an individual cope in the same manner regardless of what he or she is faced with, or are the coping strategies situation-specific? Is coping trait or state?

According to Lazarus and Folkman (18) coping is not a personality trait or style that remains stable across situations. Instead, coping is considered to be a set of strategies, based on a sequence of appraisals that are available to be implemented to match specific situations.

Self-efficacy:

A cognitive approach applicable to the stress and coping process is the theory proposed by Bandura (20), which claims that stress reactions are primarily a function of low self-efficacy to exert control over a stressful situation. Similar to the transaction model of stress, Bandura (20) contended that stress results when one's perceived coping resources are inadequate (similar to a secondary appraisal). The theory specifies that coping self-efficacy can be enhanced (and stress reduced) when one has mastery of experiences with comparable stressors, vicarious experiences that successfully overcome the stressor, verbal information that one can in fact successfully overcome the stressor, and a reduction in physiological arousal.

Bandura recognised the existence of parental self-efficacy and emphasised that for parents to enact parenting behaviour successfully, they must believe that their actions will have the desired outcome and have confidence in their ability to perform specific behaviours or skills (20). Parenting self-efficacy is broadly defined as an individual's appraisal of his or her competence in the parenting role (21, 22). Greater perceived competence in parenting is associated with assessing situations as less problematic and feeling confident that difficulties can be resolved (23-25).

Self-efficacy is found to be associated with breastfeeding initiation and duration (26, 27), and may help expectant parents cope with birth (28) and following discharge from the hospital.

Sense of Coherence:

Center for Intervention Research

Effective coping has also been suggested to be associated with characteristics within the individual. One important example is Antonovsky's theory on sense of coherence (SOC) (29).

Antonovsky (29, 30) proposed that SOC is an individual attribute that protects the individual against the harmful consequences of stressors. SOC is an indicator of resilience and personal strength that represents one's capacity to respond to stressful life events (30). People with a strong SOC regard their environment and the events in their everyday life as comprehensible, manageable, and meaningful. Antonovsky defined SOC as: The extent to which one has a pervasive, enduring though dynamic, feeling of confidence that one's environment is predictable, and that things will work out as well as can reasonably be expected (29). In other words, SOC is a combination of optimism and control. It has three components - comprehensibility, manageability, and meaningfulness (29). These three components are important, if people are to be able to handle stressors successfully and thus remain healthy. The individual needs to know what to do about a stressor, be able to do it and understand why.

Antonovsky emphasises three different lifelong learning processes that are interrelated. If the quality of the learning processes is good, they strengthen the individual by providing a greater sense of coherence.

- The feeling of predictability lays the foundation for comprehensibility.
- An appropriate load or strain lays the foundation for manageability.
- Participation lays the foundation for the feeling of meaningfulness.

1.6 Regimen rationale

The Newborn project is delivered using a health promoting population-based approach. The National Board of Health recommends that all expectant parents should be offered antenatal birth and parent preparation classes in small groups with the opportunity to discuss feelings and concerns related to birth and parenthood (14). It has been argued that information transfer by itself should no longer be the focus of antenatal education; rather, all health-promotion should provide opportunities for people to learn skills in order to practice desired behaviors (31). Experts suggest that educators need to become facilitators and adopt an outcomes-based approach, which shifts the emphasis from the educator to the learner and that individuals in order to learn and obtain new competencies need to interact with fellow learners and the learning environment (32). Currently antenatal classes are primarily offered in large auditoriums as lectures (3) with a minimum of interaction with the audience. The main focus of the antenatal lectures is on delivery and breastfeeding; information on parent-child attachment and other important psychosocial aspects that relate to parenthood are generally not covered.

In this trial the intervention group will receive antenatal education in small groups with the opportunity to obtain information as well as discussing feelings and concerns related to birth, breastfeeding and parenthood as recommended by the National Board of Health. The groups will be offered four 2.5. hour sessions – 3 sessions before birth and 1 session following birth. The session after birth has been included based on recommendations from newborn parents in previous studies. The timeframe for the

Center for Intervention Research

education is based on an estimation balancing time needed to cover the included subjects adequately, and what service providers deem as a sustainable service.

The control group will be offered standard care at the birth site, which consists of two antenatal lectures in auditoriums. As the potential benefit of both types of antenatal education remains largely unknown we consider this group division to be ethically justifiable.

1.7 Risk/benefits

There are no known risks to participating in the trial. We assume that participants in the intervention group will benefit from more in depth antenatal classes in small groups, however, we cannot rule out the possibility that the intervention group may experience an increase in worries about issues related to pregnancy and parenting. Participants randomised to the control group may experience some disappointment. The control group will receive standard care why we consider being randomised to the control group as ethically justifiable.

1.8 Ethical consideration

1.8.1 Ethical justification

The Capital region of Denmark aims to implement birth and parent preparation in small groups for all expectant parents. However, it is unknown if antenatal preparation in small groups is superior to standard care, what the classes should encompass, and what the cost-effectiveness of antenatal preparation in small groups is compared to large-scale preparation in auditoriums. In a healthcare system with limited resources it is important to develop an up-to-date research based antenatal preparation program and examine its efficiency and cost-effectiveness prior to implementation, in order to prevent excess obstetric procedures and use of health care, to improve newborn families' quality of life, and to meet the aim of evidence based medicine and most value for money. The project is a large scale, individually randomised trial of an antenatal parenting program addressing determinants of coping with birth, breastfeeding and parenting. The development of a comprehensive, theoretically founded parenting program provides an opportunity to test if antenatal parenting training – in line with the recommendations set forward by the Danish National Board of health - can increase parenting resources, promote short and long-term health and thriving in the child and the family and affect use of healthcare services. We hypothesise that a successful parenting program will increase parental resources, promote healthy parental behaviour, and improve health and thriving for newborn families; however, we do not know if birth and parent preparation in small groups is superior to antenatal classes held in auditoriums, why we believe the intervention to be ethically justifiable. Collaboration with service providers and leading national and international researchers and clinicians in this field has been established. Therefore, this proposed project may substantially improve the current "state-of-the-art".

1.8.2 Informed consent

All participants considered for this trial will be provided with written and oral information on this trial so that participants can make an informed decision about their participation in this trial. A consent form

Center for Intervention Research

must be signed by the participant and by the investigator seeking the consent, and participants will be informed of the possibility to withdraw their consent from the trial at any time.

All pregnant women receive a written invitation prior to their first birth clinic visit. Along with this invitation pregnant women will receive written information in form of a pamphlet about the trial. This information will also be available on a project website. At the birth clinic visit (<20th week of gestation) the woman will receive verbal information and give her informed consent or refusal of participation. A project phone line will be established where participants can contact a project member throughout the trial if she has further questions, or wishes to withdraw her consent for participation in the trial.

2. Trial objectives and purpose

The primary aim is to compare parenting resources, health and thriving, and use of healthcare services in newborn families enrolled in a research-based standardised antenatal birth and parenting program in small groups with those allocated to standard care.

Development of the program: We have developed a comprehensive, theoretically founded parenting program in collaboration with midwives, health care visitors, psychologists and family therapists, parents, and leading national and international researchers and clinicians in this field.

Process evaluation:

As the degree of implementation of a program is crucial to its ability to achieve any effect, we also aim to ensure careful implementation of the program, and to conduct a thorough process evaluation highlighting enabling factors and barriers to the implementation. Focus group interviews with service providers, group facilitators and participants will be carried out. Program fidelity e.g. whether the protocol is followed in program delivery, and how much of the intended program the participants are receiving, as well as program reach e.g. what proportions of the intended groups are participating in the program, has an impact on the effect of an intervention (33). These aspects, although very important, are rarely commented on when reporting the effect of an intervention. We will evaluate whether the protocol is followed in program delivery and how much of the intended program the participants are receiving. Participants will be asked to fill in an electronic questionnaire on-site at the end of each session. The questionnaire will highlight whether the intended subjects of the session have been covered as well as to what extent the participants found the information given useful. Group facilitators will be asked to fill in a similar questionnaire with the opportunity to explain why certain topics may have been omitted. Furthermore participant observation will be carried out during random sessions.

Non respondent/non-participation:

What proportions of the intended groups are participating in the program will be explored by examining demographic characteristics of those who declined to participate in the trial via the national registers, as well as examining characteristics of those who accepted and then did or did not show up for the

Center for Intervention Research

sessions. Interest and participation in parenting programs is likely only to reach some parents, for instance uptake may be related to socioeconomic position (13, 34). It is possible but unknown if offering a parenting program in small local groups increases the likelihood of participation by parents who may otherwise be difficult to reach.

Cost-effectiveness analysis:

Finally the incremental societal cost of the intervention will be calculated and compared to the measured outcomes in a cost-effectiveness analysis. Direct health care costs as well as productivity costs in terms of labour market participation and sickness absence. For this we will use data from the obstetric database at Hvidovre Hospital, national registers and questionnaires.

3. Trial design

3.1 Trial design

Individually randomised trial sited at a large birth clinic in the Copenhagen Capital Region, Hvidovre Hospital. Please find an overview of the trial design attached as separate file.

3.2 Randomisation

Information about women willing to participate in the project will be given to a project employee, who will perform individual web-based randomization according to a computer-generated allocation sequence with a varying block size kept unknown to the investigators, stratified for vulnerability (organized by the Copenhagen Trial Unit). The project employee will subsequently ensure that the birth site invites the selected women and their partners to the first session of the intervention. Women who are randomised to the control group will be sent a standardised letter highlighting the importance of the follow-up. The allocation of participants to the intervention will be 1:1 to the intervention and the control group respectively.

3.3 Trial intervention

Intervention: The intervention is developed using a systematic framework for health promotion program planners “Planning health promotion programs: an intervention mapping approach” (33). This systematic framework aids effective decision making at each step in intervention planning, implementation, and evaluation (33).

The intervention consists of a research-based general birth and parenting training program. The intervention will have two arms:

1) A research-based parenting program based on the recommendations from the National Board of Health and inspired by “Towards Parenthood” (35) and ”Prevention and Relationship Enhancement Program” (PREP). The intervention arm is described in more detail below.

Center for Intervention Research

2) Standard care (control group). The pregnant woman and her partner are offered two antenatal lectures in an auditorium.

The intervention arm: Groups of approximately 6-7 couples (or mothers) will meet three times during pregnancy and 1 time 5 weeks after expected due date, for the duration of 2.5 hours per session. (The aim is to create groups of 6-7 couples; however, due to fluctuations in uptake groups of 4-9 couples will be deemed acceptable). The sessions will include information and discussions about emotions and expectations related to birth and parenting, identification of social network resources, couple communication, breastfeeding, parent-child attachment, directions on taking care of a newborn, and recognising common signs of mood disorders and how to react. A patient-network website which enables parents to gain further information, communicate with other parents and consult online with a midwife and a health visitor will be created. In addition to gaining knowledge about issues related to birth and parenting, and creating an environment where parents can discuss their feelings and concerns, the program aims at enhancing expectant parents' awareness of their own resources and problem-solving strategies. Furthermore the way the groups are composited will enable participants to establish relations with other expectant parents in their local area. All sessions will be led by a midwife. The postnatal session will be conducted in collaboration with a health visitor, to increase knowledge on available resources in the local communities

3.4 Blinding

It is not possible to blind participants or service providers. Data will be blinded by a data- manager and the investigators and statistician will therefore be blinded to participants' intervention category during data analyses. Participants' intervention category will first be revealed to outcome assessors and investigators after they have drawn conclusions about outcome effects of group X versus Y.

3.5 Duration

Participants will be invited to participate in the trial at their first antenatal visit. The intervention consists of 4 sessions that last for 2,5 hours per session. The first intervention session will take place at 25 weeks gestation, followed by 2 sessions at 33 and 35 weeks of gestation, and a post-natal session 5 weeks after expected due date.

The control group are offered two lectures in an auditorium during pregnancy.

3.6 Discontinuation of individual participants

Participants will be able to withdraw from the trial at any time. Women who have a miscarriage or a stillborn child will be discontinued in the trial. The vice-chief midwife at Hvidovre Hospital will monitor occurrence of late miscarriages, stillbirths, and maternal deaths weekly and keep the project group informed.

3.7 Intervention accountability

Center for Intervention Research

Intervention development and data-collections are carried out by the Centre for Intervention Research and the National Institute of Public Health with guidance from relevant collaborators. The program will be developed with input from a working group consisting of midwives, health visitors, psychologists and family therapists, and representatives of the general public. Once completed, selected program facilitators from the birth sites will be trained on a workshop in the scope and delivery of the program by members of the working group.

3.8 Data collection

Data will be collected from both parents using web-based questionnaires at: baseline (<20 weeks gestation), 37 weeks gestation, 9 weeks after expected due date, 6 months after expected due date, 1 year after expected due date. Participants will be contacted via e-mail, when due to answer a questionnaire. Two reminders will be sent via additional e-mails, the first after a week and the second after 14 days. Where possible a member of the project group will contact non-respondents by phone a week after the final e-mail reminder.

4. Selection and withdrawal of participants

4.1 Inclusion criteria

Expectant pregnant women and their partners
≥ 18 years old
Singleton pregnancy
Due to give birth at Hvidovre Hospital.
Able to speak and understand Danish
Being legally able and willing to provide signed consent

4.2 Exclusion criteria

Not providing signed informed consent

4.3 Participant withdrawal

Participants are free to withdraw entirely from the Newborn trial at any time, and this will not have consequences for the participant's further treatment. When possible, participants will be asked if they will allow their already collected data to be used in a database, and/or publication.

5. Experimental interventions

A detailed intervention manual will be created for the trial facilitators. The following subjects will be covered in the sessions:

The NEWBORN programme includes short verbal presentations from the group facilitator, individual exercises, short film presentations, time for discussions and reflection. Parents are given homework in the form of minor exercises in preparation to each session. Educational subjects are: the transition to

Center for Intervention Research

parenthood; couple communication; birth; breastfeeding; and taking care of a newborn. A patient-network website has been created as a supplement to the sessions. The programme is focused on parenting resources important to the birth process, parenting and mental health, and that appear amenable to change, i.e.: social support, parenting alliance and communication with partner, cognitive coping, e.g., self-efficacy and parenting skills. These elements have been addressed in the following manner:

I. Social support: formal and informal, emotional, informational and instrumental. Groups of 6-8 couples are offered three times 2.5 hour sessions during pregnancy and one session five weeks post-partum. The groups are composited to enable participants establish relations with other expectant parents in their local area. Sessions are led by a midwife and the postnatal session will include a health visitor. A patient-network website enables parents to gain further information, communicate with other parents and consult online with a midwife and a health visitor.
II. Parenting alliance: adding a component supporting the couples in the transition to parenthood and couple communication.
III. Cognitive coping: embedding sources of self-efficacy into programme content and delivery, and by creating an environment which enables parents to discuss feelings and concerns, enhances their awareness of own resources, problem-solving strategies, and future challenges in parenting and emotional regulation
IV. Parenting skills: increasing information and exercises with feedback, e.g., on recognising signs and symptoms of thriving in the newborn, couple communication etc.

In short, the approach aims at strengthening relationships and improving information and problem solving skills for expectant parents in order to ease birth and the transition to parenthood.

To maximise the potential for population uptake classes have been established at three local midwifery sites. A comprehensive guide and education material for course facilitators has been developed, and facilitators, i.e. midwives and health visitors are trained at one-day workshops. The framework for the classes is based on an estimate of adequate time allocated to each subject, and what service providers deem a sustainable service.

Center for Intervention Research

<u>Session 1 (approximately 25 weeks gestation):</u>

- The transition to parenthood – new roles and responsibilities, emotional adjustment
- Common changes and challenges in the relationship during and after pregnancy
- Couple communication

- Meaning of own childhood when becoming parents

During the first session the parents are introduced to one another and to the scope and outline of the entire programme. The midwife invites the parents to think about and since discuss their expectations of the greatest joys and greatest challenges of parenthood. Afterwards the midwife informs the parents about common changes and challenges in the relationship during pregnancy and after birth, and the importance of good communication. A short film is shown teaching the parents about good communication skills. The film is developed by the Danish Centre of Family Development and is inspired by PREP (36). The film is followed by practical couple-communication exercises. Women attending the classes alone either pair up or conduct the exercise with the midwife. In short the exercise entails one person listening actively and without interrupting while the other person describes thoughts and feelings about a certain topic. Afterwards the person listening is encouraged to acknowledge what she/he has heard before changing roles. This communication exercise is used throughout the entire programme covering different topics e.g. expectations of parenthood, labour, the relationship after birth etc. The aim is to try to understand the other person's perspective before trying to be understood. The importance of one's own childhood when becoming a parent is also a topic in this session (35). Participants are asked to think back to their own childhood, how they were raised, their parents' parenting style (e.g. warm and affectionate, strict etc), traditions etc. Afterwards they are to consider things they would like to carry forward into their own parenting as well as things they might want to do differently. Finally the couple begin to discuss the topic using the communication technique they have been taught. The aim is to start a thought process. As there is not sufficient time for long in depth discussions participants are encouraged to carry on the discussions at home. During the first break participants who wish to do so are asked to write down their contact details so that they can be shared in the group. Throughout all the sessions the midwife has a facilitating role helping discussions along if needed and commenting where appropriate. At the end of all sessions participants are asked to consider and write down the most important take home points of the session.

Suggested preparation for the next session: seven short informational film clips (duration between 2 and 7 minutes) on the first signs of labour, the time at home in early labour, birth, when there is a need for obstetric intervention, and pain relief.

The films, exercises, and written information on session topics are available on the network website.

Session 2 (approximately 33 weeks gestation):

- Expectations in relation to birth

Center for Intervention Research

• The normal course of labour
• When there is a need to intervene in labour
• Pain relief and coping strategies
• Partner support during labour

The aim of this session is providing the participants with information, and enhancing their existing knowledge and understanding of the normal course of labour, pain-relief, and what might be expected if there is a need for obstetric intervention. After a short verbal presentation by the midwife the couples discuss their hopes and expectations for labour and birth using the communication framework - they are asked to consider their individual resources and action competencies in relation to increasing the likelihood of obtaining their wishes. Also the couples are asked to discuss how they might support one another during birth and labour – using practical examples. For the topic on pain relief the women are first asked to discuss their thoughts and previous experiences with coping with pain and physical and mental strain – what did they do, what helped them, can they use any of these strategies during labour? Next they discuss their thoughts and knowledge on various methods of pain relief. Meanwhile the men discuss their thoughts and feelings about their role during labour and birth. Plenary discussions and summing up thoughts and ideas are used so that participants can learn from and be inspired by one another. Vicarious learning and feedback are considered important in relation to self-efficacy (20).

Suggested preparation for the next session: participants are encouraged to ask women in their social network about their breastfeeding experiences, and read a pamphlet that is handed out on breastfeeding (37).

<u>Session 3 (approximately 35 weeks gestation):</u>
• Feeding a newborn –including breastfeeding intention, expectations, facts and myths
• Interpreting the newborn’s signs, symptoms and behaviour
• Taking care of a newborn – baby cues, bonding, and practical issues
• Mood swings and postnatal depressive symptomatology

Participants discuss wishes for feeding their newborn and feeding experiences in their networks in small groups. The midwife then talks about how expectations, support, and the advice received from family and friends may affect e.g. breastfeeding intention and perseverance in the case of difficulties. Bearing the breastfeeding experiences of individuals in their social networks in mind (preparation for this session), participants are encouraged to consider who it might be most helpful to seek breastfeeding support and advice from if necessary.

Center for Intervention Research

Cards with a variety of breastfeeding topics are spread out on the table, and participants are asked to pick a topic that they wish to hear more about, and tell the group why they have picked the chosen topic. Topics include e.g. how to tell that the baby is getting enough milk, positioning, importance of partner support, feeding patterns, breast engorgement etc. There are certain topics that the midwife is told to cover regardless of whether it has been picked by a participant or not e.g. how to tell the baby is getting enough milk. The pilot study showed that participants are likely to choose a topic they already know something about in order to receive verification and feedback from the midwife and from the other participants (unpublished data) – this may help increase self-efficacy.

The midwife gives information and shows short film clips on baby cues and sleep patterns. The importance of communicating with the newborn is underlined. Information on the prevention of cot death is given. Next the initial time at home with a newborn and the importance of social networks for emotional and practical support is discussed (the group is considered a potential supportive social network). Participants are given an exercise where they are asked to fill in a list of expected daily activities after the baby is born. Afterwards they compare their list with their partner's or that of another group member before summing up in plenum. The aim of this exercise is to increase awareness of what changes life with a newborn has on a daily routine, how much time is spent on breastfeeding etc.(35). Participants are also asked to consider activities that give them energy and pleasure (e.g. playing football, going out with friends, reading a book), and how they might incorporate some of these activities in their new daily lives (35). Next participants are encouraged to reflect upon how they normally handle worries, and to discuss this topic with their partner. Finally common emotional reactions and postnatal depressive symptomatology is covered. The importance of being open about these emotions and supportive of one another is stressed, as is the importance of seeking help when deemed necessary.

<u>Session 4 (approximately 5 weeks post-partum):</u>

• Birth experiences
• Mood swings and postnatal depressive symptomatology
• The first time at home with a newborn – experiences, challenges and solutions
• Couplehood – partner support, communication, division of household tasks

This session is carried out by a midwife as well as a healthcare visitor. The aim is for the newborn parents to share birth experiences, and their experiences in their new roles as parents so far. The topic of common emotions and postnatal depressive symptomatology is revisited. Next groups of four are asked to discuss how being a parent is different to what they expected, which challenges they consider to be the greatest, how they cope with/handle these challenges, and what have been the greatest joys. The parents are able to ask the midwife and the healthcare visitor practical questions during the break.

Center for Intervention Research

After the break, using the communication framework, couples are asked to discuss what the best change has been in their partner after becoming a parent, what has worked really well in the relationship, and in sharing household tasks, and what could make it even better. Finally the healthcare visitor talks about sex (including contraception) and intimacy after becoming parents.

5.1 Concomitant medication/treatment

All participants are free to make use of concomitant antenatal/postnatal services and parent groups. As use of other services and parent groups may influence the outcomes that we are interested in, use and services will be examined, documented and given careful consideration in the analyses.

5.2 Monitoring for participant compliance

We will evaluate whether the protocol is followed in program delivery, how much of the intended program the participants are receiving, as well as what proportions of the intended groups are participating in the program. Furthermore we will monitor use of the website.

6. Assessment of outcome measures

6.1 Efficacy variables

Data will be collected via the hospital obstetric database, the national registers, and web-based questionnaires and collected in an electronic case record form (eCRF) at: baseline i.e. time point 0 (tp0) (<20 weeks gestation), 37 weeks gestation (tp1), 9 weeks (tp2), 6 months (tp3) and 1 year after expected due date (tp4).

<u>The primary outcome is:</u>

Epidural use during labour – data from the hospital obstetric database (tp2)

<u>Secondary outcomes are:</u>

Stress – perceived stress, parenting stress - questionnaire data (tp0,2,3,4)

Parenting alliance – questionnaire data (tp2,3,4)

<u>Explorative outcomes:</u>

Antenatal and Post-natal depressive symptomatology and anxiety – questionnaire data (tp0,1,2,3)

Breastfeeding - questionnaire data (tp0,1,2,3)

Use of healthcare services i.e.: for the parents obstetric intervention i.e. augmentation of labour, vacuum extraction, caesarean rate - data from the hospital obstetric database (tp2), and contact to

Center for Intervention Research

healthcare professionals for depressive symptomatology and anxiety, and unscheduled postnatal visits– questionnaire data (tp2,3). For the child i.e. neonatal readmissions to hospital, contacts to accident and emergency departments (A & E), GP and doctor on call during the child's first year of life (composite measure). Also use of the regional emergency phone line – data from the national registers (tp4).

Mental well-being – questionnaire data (tp 1,2,3)

Family medicine use – questionnaire data (tp2,3) and register data (tp4)

Smoking - questionnaire data (tp 1, 2)

Satisfaction with relationship and family break-ups - questionnaire data (tp 0,2,3), and data from the national registers on divorce and break-ups (tp4)

<u>In this trial the intermediate outcomes of interest are:</u>

Parenting resources: Trust in own ability to cope with/self-efficacy: 1) birth (tp1), 2) discharge (tp1) 3) parenting (tp2,4) breastfeeding (tp0,1); couple communication (tp0,1,2,3); social support/network (tp0,1,2,3)

Measurements – background, tools and discussion

Being prepared for childbirth and parenthood and having available resources for meeting the demands may help parents cope with many concomitant situations.

In trials with subjective outcomes effect estimates are often exaggerated when there is inadequate or unclear allocation concealment (38). Blinding of participants and midwives is not feasible in this trial. In this situation it is desirable to specify at least one objectively assessed outcome, even if the outcome of most interest is subjective (38).

<u>Primary outcome:</u>

Epidural use during labour – using data from the hospital obstetric database (Birth)

Findings suggest that women who receive epidural analgesia experience more fear but not more pain, before the administration of epidural analgesia (39). Structured antenatal education may improve women's ability to cope during labour resulting in lower epidural rates (28).

<u>Secondary outcomes:</u>

Stress:

The perceived stress scale (PSS) will be used to measure global levels of perceived stress (tp 0,1,2,3,4). The PSS takes into account the individual's ability to cope (40). The scale was developed on the basis of appraisal theory (18) and was designed to " . . . tap the degree to which respondents found

Center for Intervention Research

their lives unpredictable, uncontrollable, and overloading" (40). The PSS is widely cited, has been used in several studies of life stress, and has been translated into several languages. The PSS has been found to predict many adverse health outcomes (41). There has been some concern that psychological symptoms and the PSS may overlap in item content (40). However, the PSS has been shown to prospectively predict health outcomes independent of psychological symptoms (40, 42), supporting the discriminate validity of the PSS with regard to psychological symptoms (43). Despite limitations inherent in stress response measures and cautionary concerns about confounding with psychological symptoms, overall the PSS possesses good psychometric qualities and a respectable record of validity studies (41).

Parenting stress will be measured using *the Swedish Parenthood Stress Questionnaire* (SPSQ) (44) translated into Danish (tp2,3,4). The scale takes into account the individual's ability to cope and assesses parental stress with a sum score and subscale scores of incompetence, role restriction, social isolation, spouse relationship and health problems (44). Results from SPSQ have been related to physiological and psychological health, and socioeconomic and lifestyle factors (45). Furthermore significant correlations between SPSQ and clinical assessments of maternal stress and parental problems in responding to their child have been found (45).

Parenting stress has been shown to relate to negative child, parent, and family aspects e.g. lack of maternal emotional availability (46), maternal hostility (46), insecure child attachment (47, 48); parents' use of undesirable managerial techniques, (49) and more strict disciplinary style (50); child abuse and neglect, (51); depression in mothers, (52, 53), and poor marital quality (54).

Parenting alliance

Parenting alliance will be measured by the Parenting Alliance Measure (tp3,4) (55). It is believed that this is a more specific measure to the prediction of parenting behaviors than marital satisfaction because it recognizes that both parents can be involved, and function well, in the parenting role, and yet not be very satisfied with their personal relationship with each other (2, 55).

<u>Explorative outcomes:</u>

A health promoting antenatal program enhancing early parenting resources may affect a variety of outcomes in a positive way, however, due to the scope of this trial and power considerations the following outcomes will be explorative in nature.

Stress frequency and stress intensity

Two single item questions on perceived stress capturing how often an individual feels stressed and to what extent will be included (tp0,1,2,3,4). The questions are validated in a Danish population and derive from the Longitudinal Copenhagen City Heart Study (56).

Antenatal and postnatal depressive symptomatology and anxiety

Center for Intervention Research

In order to identify mothers (and fathers) with a possible emerging antenatal or postnatal depression, we will use the 10-item Edinburgh Postnatal Depression Scale (EPSD) (tp 0,1,2), and the Major Depression Inventory (MDI) (ICD-10) (tp 3). The EPDS is currently believed to be the best non-comprehensive screening tool, despite its relatively low predictive value and has recently been evaluated in a Danish population (57). The MDI is used to increase the diagnostic specificity and has similarly been used in a Danish population. The Hopkins Symptom Check List (SCL-25) first 10 items (anxiety score, SCL-anxiety) will be used to measure anxiety (tp 1,2,3).

An estimated 7% of expectant mothers suffer from depressive symptoms during the first trimester of pregnancy, increasing to 12-13% during the third trimester and 3 months postpartum (58, 59). Depression is associated with a high risk of anxiety, poor self-care, partner relationship problems (60), fear of childbirth (61), and poor obstetric outcomes (62). Furthermore maternal depression is associated with impaired mother-infant relations (63), an increased risk of feeding and growth problems (64), and regulatory and emotional problems (65-71) in the child.

The Newborn trial aims to address some of the risk factors associated with AND and PND e.g. self-efficacy, stress and social support (72, 73) which may reduce the occurrence. However, depression is a complex phenomenon with many pathways. As PND may have negative long-term consequences - especially if left untreated - how to recognize signs and symptoms of PND and how to act on these symptoms (e.g. contacting the general practitioner) is included as an important topic in the Newborn trial. It is therefore possible that a larger proportion of the intervention group will report and seek help for AND and PND.

Breastfeeding:

We will measure the following factors in relation to breastfeeding: intention (tp0,1), trust in ability to cope with breastfeeding (tp0,1), difficulties (tp2), partner support (tp0,2), duration of exclusive breastfeeding (tp3) by single items specifically designed for this study. Feeding intention, knowledge, self-efficacy and social support are important determinants for breastfeeding initiation and duration (74), and breastfeeding is an important topic in the antenatal education in the present trial.

Use of healthcare services:

Among the parents use of health care services will be explored by examining: obstetric interventions i.e. augmentation of labour, vacuum extraction and caesarean section using data from the hospital obstetric database (Birth). Women who spend less time at home in early labour before presenting at the hospital are more likely to have an obstetric intervention (e.g. caesarean section) than those presenting in more advanced labour (75). Structured antenatal education may improve women’s ability to cope at home in early labour as well as during labour resulting in less obstetric intervention (28). Additionally parental contacts to healthcare professionals for postnatal depressive symptomatology (tp2,3), and unscheduled health care contacts post discharge - i.e. contacts initiated by the mother for purposes other than a

Center for Intervention Research

scheduled, planned follow-up with her own health care provider for usual postnatal follow-up care - will be measured using questionnaire data (tp2).

Use of healthcare services for the child: neonatal readmissions, contacts to A&E, GP, and doctor on call in the national registers (tp4). Due to power considerations this measure will be a composite measure. Each component will also be analysed separately. According to service providers insecure parents use the healthcare services beyond indication. Janicke and Finney developed an efficacy-related model of paediatric services, suggesting that the use of these services is a function of perceived parental stress and low self-efficacy in coping with life demands (76). The Copenhagen Capital region has established an emergency phone line, and citizens are encouraged to use this when in doubt about appropriate course of action in relation to illness and accidents. Use of the emergency phone will be examined in the national registers (tp4). A program to increase knowledge on newborn care, parental self-efficacy and social network and support may affect use of healthcare services.

Satisfaction with relationship and family break-ups

Satisfaction with relationship will be measured with a scale from the Avon Longitudinal Study of Parents and Children (ALSPAC) (tp0,1,2,3,4) (77). Furthermore we will examine divorce and break-ups within the child's first year of life using the national registers (tp4).

The trajectory of marriages/relationships following the birth of a child is likely to have important implications for both long-term health of the parent's relationship and their children's development (78). Social support is important during pregnancy and childbirth and the partner is usually the main source of support. A Swedish cohort study found a higher rate of divorce among women who were unsatisfied with their partner's support during pregnancy (79). Early family transitions that strain couple relationships provide opportunities for preventive interventions to strengthen marriage (78). In the Newborn trial there is a focus on couple communication and expectations, and the importance of partner support and mutual respect and understanding.

Medicine use

Family medicine use will be recorded by use of questions from the Danish National Health survey program (tp2,3,4) (80). Some medicine is sensitive to psychosocial circumstances and may reflect a general strategy for coping with daily stressors (81-83) . A high level of perceived stress is associated with use of over-the-counter medicine, primarily analgesics (82-84). Depression is also associated with excessive analgesic use (84). Administration of over the counter medications, especially analgesics, for children is common (85). Not all medicine use among children can be explained by prevalence of symptoms (85). Qualitative studies suggest that some medicine administration may be a form of "social medication," to give parents control over children's behavior that they perceive as fractious and irritating (86)

Center for Intervention Research

Smoking is associated with stress and will be measured by questions from the national health interview surveys.

Mental well-being: Mental health promotion and wellbeing strategies are urgent public health concerns and the need for the promotion of mental health and wellbeing has now become a feature of government policy within many countries throughout the world. It is possible that the Newborn intervention may increase mental wellbeing. It will be measured using the Warwick-Edinburgh mental well-being Scale (WEMWBS) (tp 1,2,3,4).

Intermediate measures:

When evaluating health promoting programs a number of intermediate indicators (e.g. self-efficacy, beliefs, knowledge, skills acquisition) should be used to asses various components of a program and to allow illuminative evaluation (87).

Furthermore for some particular health promotion models, intermediate indicators will serve as outcome indicators (87). For example, while evidence of self-efficacy might provide an intermediate indicator of ultimate success in terms of a reduction in risk factors for a negative outcome, self-efficacy might equally be judged as a desirable outcome in its own right for a salutogenic model of health promotion.

Central to how parents adapt to parenthood are their perceptions of having access to available resources for meeting the demands of parenthood (2, 11). Some of the most important resources related to parenting are: self-efficacy, social network and support, and parenting alliance (2, 23, 88). The Newborn antenatal education program aims to increase these resources, hence the transition to parenthood should be smoother and less stressful if the program is successful.

Trust in own ability to cope with birth

Will be measured using questionnaire data (tp1). Questions are developed for this trial. Structured antenatal education may improve women's trust in their ability to cope with birth resulting in e.g. lower epidural rates (28).

Trust in own ability to cope at discharge

Will be measured using questionnaire data (tp1). Questions are developed for this trial. Perceived readiness for discharge has become increasingly important to asses as hospital length of stay following birth has decreased - it may affect patient satisfaction, physical, emotional , psychological, and social outcomes (89).

Parenting self-efficacy (PSE)

To evaluate parenting self-efficacy (PSE) i.e. the individual's appraisal of his or her competence in the parenting role Salonen's PSE scale measuring parents' perceived competence in day-to-day infant care tasks, as well as interactive behaviour will be used (tp 2) (90).

Center for Intervention Research

High PSE is important for sense of well-being and parenting satisfaction (23, 91). PSE has been identified as an important buffer against parenting stress (23, 88), a major determinant of parenting behaviors, and closely linked to child development outcomes and psychosocial child adjustment (25, 92-94). Experience of childbirth has an impact on PSE (95-97). Findings suggest that parenting programs may improve PSE (98-101). However, studies on PSE around the time of childbirth are scarce (90, 100, 102-104), hardly any are randomised trials, sample sizes are small, and thus generalisability of findings is limited (100, 102).

Trust in own ability to breastfeed

Will be measured using questionnaire data (tp0,1). Two questions are inspired by Kronborg (26) and further developed for this trial. Trust in own ability to breastfeed is associated with breastfeeding initiation and duration (26, 27).

Couple communication

Couple communication will be measured with a scale from the Avon Longitudinal Study of Parents and Children (ALSPAC) (tp0,1,2,3,4) (77). Identifying effective ways of talking and listening is likely to improve the quality of couple communication, which in turn may increase parenting alliance and satisfaction with the relationship.

Network and social support

Social support (structure and function) will be measured by two previously well validated questions from the Danish Longitudinal Health Behaviour Study (tp0,1,2,3,4) (105) as well as by 6 questions designed for this trial (tp1,2).

Based on the literature the theoretical definition of social support for this trial is the combination of social structures and social functions, where social structures demonstrate cohesiveness and there is a flow of emotional concern, instrumental aid, information and appraisal between people (73). Functional social support has four dimensions: informational, instrumental, emotional and appraisal support. Structural social support has two dimensions: formal (health care professionals) and informal (family, friends and significant others) (73). The Newborn trial addresses all these dimensions of social support.

Previous research has shown that social support can facilitate women's transition to motherhood (106, 107), some of whom find the transition psychologically stressful (108).There is evidence that social support from staff and different kinds of hospital practices have a positive impact on parenting (97, 109, 110) In a Swedish study women dissatisfied with partner support during pregnancy experienced more physical symptoms, more depressive symptoms, more major worries and a lower sense of coherence. One year after childbirth a higher rate of divorces was found in women being dissatisfied in early pregnancy (79). Social support for first-time mothers may play an important part in both enhancing maternal parental self-efficacy and reducing postnatal depressive symptomatology in the early postpartum period (73)

Center for Intervention Research

Demographic variables and individual characteristics:

Education: In this trial, education is assessed using the International Standard Classification of Education (ISCED) (UNESCO 1997) which combines school and vocational education (tp0).

Occupational social class: The coding format used to define occupational social class in this trial is based on the coding principles used by the National Danish Centre for Social Research for many years. This format is in accordance with the social class classification of the Registrar General in the UK. The format is a five- point classification scheme (I-V). Individuals are classified based on occupational position, educational qualifications required and the number of subordinates. Individuals outside the labour market will be classified as occupational social class VI (tp0).

Marital status, cohabiting status and amount of children living in the household will be measured using standard health survey questions (tp0).

Sense of coherence will be measured using the 9 item scale developed by Setterlind and Larsson (111). This is a validated scale previously used in Danish population-based surveys as well as in a prospective survey among Danish women receiving fertility treatment (tp0,3) (112).

Self-rated health – physical and emotional (tp0,2,3,4), subjective health complaints (0,2,3,4), and long term illness (tp0) will be measured by well validated questions from the Danish National Health survey program (80).

Measures of program implementation and use of additional antenatal and postnatal services:

We will evaluate whether the protocol is followed in program delivery and how much of the intended program the participants are receiving. Qualitative interviews will be conducted with selected participants and program facilitators. Also participants will be asked to fill in a questionnaire following each session highlighting whether the intended subjects of the session have been covered. Group facilitators will be asked to fill in a similar questionnaire with the opportunity to explain why certain topics may have been omitted.

Measures of non-response/non participation: What proportions of the intended groups are participating in the program will be explored by examining demographic characteristics of those who declined to participate in the trial via the national registers, as well as examining characteristics of those who accepted and then did or did not show up for the sessions.

Use of additional antenatal and postnatal services will be examined via questions specifically developed for this purpose.

Outcome assessment committee

The reading and interpretation of the outcomes will be centralized at the Centre for Intervention research and the assessor will be blinded of the participants' intervention group.

Center for Intervention Research

6.2 Method and timing

Table 1: Assessment tools and outcome measures

Baseline is time point 0 (tp0) (<20 weeks gestation), 37 weeks gestation (tp1), 9 weeks (tp2), 6 months (tp3) and 1 year after expected due date (tp4).

Tools	Outcomes	Time points
Primary measure		
Hvidovre hospital obstetric database	Epidural use	• Birth
Secondary Measures		
Perceived stress scale	Stress	• 0,1, 2,3,4
Swedish Parenthood Stress Questionnaire (SPSQ)translated into Danish	Parenting stress	• 2,3,4
The Parenting alliance measure	Parenting alliance	• 3,4
Explorative Measures		
Single item question from the Longitudinal Copenhagen City Heart Study	Stress frequency	• 0,3,4
Single item question from the Longitudinal Copenhagen City Heart Study	Stress intensity	• 0,3,4
10-item Edinburgh Postnatal Depression Scale	Antenatal and Postnatal depressive symptomatology	• 0,1,2
MDI, ICD-10	Depressive symptomatology	• 3
The Hopkins Symptom Check List (SCL-25) first 10 items (anxiety score,SCL-anxiety)	Anxiety	• 1,2,3

Center for Intervention Research

**Tools**	**Outcomes**	**Time points**
Single item	Breastfeeding (intention)	• 0,1
Single item	Partner support in relation to breastfeeding	• 0,2
Single item	Breastfeeding (difficulties)	• 2
Single item	Breastfeeding (duration)	• 3
Hvidovre hospital obstetric database	Use of healthcare services (Obstetric intervention)	• Birth
Danish national Registers	Use of healthcare services	• 4
Single item question	Use of healthcare services for postnatal depressive symptomatology	• 2,3
Single item question	Use of healthcare services - maternal unscheduled health care contacts post discharge	• 2,3
Scale from ALSPAC	Satisfaction with relationship	• 0,1,2,3,4
Danish national Registers	Divorce/break-ups	• 4
Medicine use question from the National health interview survey	Medicine use	• 2,3,4
Danish national Registers	Medicine use for depression	• 4
Smoking question from the National health interview survey	Smoking	• 1,2,3,4
Mental Well-being	The Warwick-Edinburgh Mental Well-being Scale	• 1,2,3,4
Intermediate measures		

Center for Intervention Research

**Tools**	**Outcomes**	**Time points**
Single item questions	Trust in own ability to cope with birth	• 1
Single item questions	Trust in own ability to cope at discharge	• 1
Two single item questions	Trust in own ability to breastfeed	• 0,1
Scale from ALSPAC	Communication in the relationship	• 0,1,2,3,4
Two questions from the Danish longitudinal Health Behaviour Study	Network and perceived social support – informal: emotional and instrumental	• 0,1,2,3,4
Six questions	Network and perceived social support – formal : informational	• 1,2
Demographic and individual characteristics		
ISCED	Education	• 0
Five point classification scheme - questionnaire	Occupational social class	• 0
Four questions	Marital status, cohabiting status, children living in the household	• 0
SOC 9-item scale by Setterlind and Larsson	Sense of coherence	• 0,3
Two single items	Self-rated health (Physical/emotional)	• 0,2,3,4
Questions from the National health interview survey	Subjective health complaints	• 0,2,3,4
Questions from the National health interview survey	Long term illness	• 0

Center for Intervention Research

7. Statistical plan and data analysis

7.1. Sample size and power estimations

Previous RCTs on antenatal parenting interventions have been difficult to interpret partly because of problems related to small sample sizes. This trial will to our knowledge be the biggest RCT on an antenatal parenting intervention to date.

Sample size

Previous data from trials (28) and hospital databases (113) indicate that the proportion of women who use epidural analgesia during birth is between 23 and 41 %. The proportion is higher among first-time mothers. Assuming that the majority of included participants in the NEWBORN trial will be first-time mothers, it is plausible to assume a proportion of epidural use of 31 % in the control group. If use of epidural analgesia can be reduced from 31 % in the control group to 25 % in the experimental group (risk reduction of 16.7 %), with a type I error (alpha) of 5 % and a type II error of 20 % (power of 80), then this requires randomisation of a total of 878 women to the intervention group and 878 women to the control group.

Power estimation for the secondary outcome Perceived Stress Scale

We are planning a trial with 878 experimental participants and 878 control participants. In a previous study the Perceived Stress Scale response within each participant group was normally distributed with standard deviation 6 (114). If the true difference in the experimental and control means is 1, we will be able to reject the null hypothesis that the population means of the experimental and control groups are equal with probability (power) 93.7%. The type 1 error probability associated with this test of this null hypothesis is 5%.

Power estimation for the secondary outcome Swedish Parenthood Stress Questionnaire

In a previous study the Swedish Parenthood Stress Questionnaire response within each subject group was normally distributed with standard deviation 0.5 (44). If the true difference in the experimental and control means is 0.1, we will be able to reject the null hypothesis that the population means of the experimental and control groups are equal with probability (power) 98.7%. The type 1 error probability associated with this test of this null hypothesis is 5%.

Power estimation for the secondary outcome Parenting Alliance Measure

In a previous study the Parenting Alliance Measure response within each subject group was normally distributed with standard deviation 20 (55). If the true difference in the experimental and control means is 4, we will be able to reject the null hypothesis that the population means of the experimental and control groups are equal with probability (power) 98.7%. The type 1 error probability associated with this test of this null hypothesis is 5%.

Center for Intervention Research

7.2 Statistical methods

Reporting will follow the guidelines of the CONSORT-statement. Statistical analyses will be intention to treat as well as per protocol. The level of significance will be 0.05.

The analysis of the primary binary outcome will be done using the generalised linear mixed model with distribution = binomial, link = logit.

If the percent missing cases > 5%, the results of this analysis will be subjected to a 'worst case' and a 'best case' scenario analysis of the potential impact of missing values. Assume a beneficial effect (less use of epidural analgesia) is noted in one group (group A) as compared to the other group (group B). A worst case scenario will then be constructed where missing values in group A are imputed by a "yes" to use of epidural analgesia and missing values in group B are imputed by a "no" to use of epidural analgesia. A corresponding best case scenario will also be constructed and the result under both scenarios will be computed.

Analyses (adjusted by baseline value and protocol specified variables) of perceived stress at 37 week gestation and for each of the other two secondary outcomes of the area under the curve (AUC) from 9 weeks to one year after due date will be done.

If the percent missing cases of an outcome is >5% and p of Little's test (1) < 5%, a number of datasets with observed values and predicted unobserved values necessary to produce an efficiency > 99% will be produced using multiple imputations (SAS version 9.3 or later)(2). The primary analysis will then be that based on these data sets.

Multiplicity will be dealt with as follows: the primary outcome will be tested at the 5% level. The p values of the remaining four tests will be adjusted using Hommel's procedure (Konietschke, 2012). In all events all observed p values will be reported.

For the secondary outcome measure 'Perceived Stress Scale' it will be tested using the general linear univariate model, whether the mean values differ between the two intervention groups at time 1 (37 week gestation), time 2 (9 weeks following birth), time 3 (6 months following birth), and time 4 (12 months following birth).

For the secondary outcome measure 'Swedish Parenthood Stress Questionnaire' it will be tested using the general linear univariate model, whether the mean values differ between the two intervention groups at time 2 (9 weeks following birth), time 3 (6 months following birth), and time 4 (12 months following birth).

For the secondary outcome measure 'Parenting Alliance Measure' it will be tested using the general linear univariate model, whether the mean values differ between the two intervention groups at time 3 (6 months following birth), and time 4 (12 months following birth).

Center for Intervention Research

The unadjusted analyses are the primary analyses. If possible all analyses will be repeated adjusted for the protocol specified stratification variable and the baseline value.

Three explorative subgroup analyses may be conducted in each case provided the subgroup interacts significantly with the intervention.

In addition to analyses performed for individuals, descriptive analyses reporting the interdependency within the couple as well as within the specific antenatal education group, and at the facilitator level will be performed by intraclass correlation.

Process evaluation will be conducted with a mixed methods approach using quantitative questionnaire data and qualitative interviews with participants and service providers. We will examine program fidelity, e.g. whether the protocol is followed in program delivery, and how much of the intended program the participants are receiving, as well as program reach e.g. what proportions of the intended groups are participating in the program, as these factors have an impact on the effect of an intervention(33). The incremental societal cost of the intervention will be computed and compared to the measured outcomes in a cost-effectiveness analysis. Direct health care costs and productivity cost in terms of labour market participation and short term absence will be calculated.

7.3 Significance

Significance level is set to 0.05 and power to 0.80. See 8.1

8. Direct access to source data/documentation

The trial will be carried out in accordance with the Declaration of Helsinki in its latest form as well as national laws and regulations.

The investigators permit audits and inspection by providing direct access to source data/documentation

The trial will be monitored by an unrelated research group at the National Institute of Public Health. eCRFs will be checked, and as minimum the following will be monitored locally:

All patients for existence (If the CPR number is correct)
All patients for documented informed consent

9. Data handling and record keeping

All participant data are protected in accordance with the Danish Act on processing of personal data and the Danish Health Act. Source data will be registered in the participant's eCRF. A common web-based electronic case record form (eCRF) will be devised to enable a central database. Data entry into the central database and handling of data is the responsibility of the investigators. Data will be stored in accordance with guidelines issued by the Danish Data Protection Agency, with which the trial is also

Center for Intervention Research

registered. The database will be kept according to the respective national laws. After the end of the trial, the data will be archived for five years according to good clinical practise guideline.

Data will be collected from both parents using web-based questionnaires and collected in an electronic case record form (eCRF) at: baseline (<20 weeks gestation), 37 weeks gestation, 9 weeks, 6 months, and 1 year after expected due date. The CRFs will be designed by a Data manager at the National Institute of Public Health.

Informed consent will be kept in paper form and stored in accordance with guidelines issued by the Danish Data Protection Agency.

After completion of statistical data analysis, data will be pseudo-anonymised and deposited at the Danish Data Archive. Also, data will be made available for other researchers by uploading a completely anonymised dataset onto Clinicaltrials.gov.

10. Quality control and quality assurance

11. Trial organisation

The project will be led by:

- Pernille Due, Director of the Child Health Program at The National Institute of Public Health (NIPH), University of Southern Denmark, Child Work Package Leader at the Centre for Intervention Research (CIR)
- Morten Grønbæk, Director of (NIPH), Scientific Chair of CIR
- Vibeke Koushede, Principal Investigator, Researcher at the NIPH and CIR
- Carina Brixval, Research Assistant at the NIPH and CIR
- Solveig Forberg Axelsen, Research Assistant at the NIPH and CIR

In close collaboration with an expert group representing key scientific competencies:

- Christian Gluud, Head and founder of The Copenhagen trial Unit
- Tom Weber, Chief of obstetrics, consultant obstetrician and gynecologist, Hvidovre Hospital
- Marianne Skovby, Chief Midwife at Hvidovre Hospital
- Poul Videbæk, Professor at the Centre of Psychiatric Research, Aarhus University Hospital
- Gerjo Kok, Professor of Applied Psychology, Maastricht University, The Netherlands
- John Lynch, Professor of Public Health at the University of Adelaide, Australia
- Naja Hulvej Rod, Head of the Copenhagen Stress Research Centre

For detailed program planning and development of the intervention, the expert group will be supplemented with an interdisciplinary group of practitioners: Obstetricians, midwives, health visitors, psychologists, family therapists, and members of the patient organisation 'Parenting and Childbirth'. (Once completed, selected program facilitators from the birth sites will be trained on a workshop in the scope and delivery of the program by members of the working group).

Center for Intervention Research

Together the groups bring to the project their expertise in public health science and epidemiology, preventive medicine, psychology and pedagogy; design and management of health-promotion interventions and randomised clinical trials; management of antenatal and postnatal services; and it-solutions.

NIPH and CIR will be in charge of the intervention development, data-management, and statistical analysis. The trial protocol will be developed in collaboration between NIPH and CIR and CTU. Randomisation and statistical analysis plan will be organised by CTU. The Centre of Family Development will help develop course material. Recruitment of participants, organization of facilities, and scheduling of program sessions will be carried out by Hvidovre Hospital. The Danish e-Health Portal will create a website in line with their existing websites for patient organisations.

Rambøll Management Consulting will develop a specialised it-system that can handle the complexity of multiple data-collections carried out at individual time-points, and a system to collect on-site data relevant to process-evaluation.

12. Legal aspects

12.1 Finance and insurance

The Danish Cancer Society have funded the project with 5,2 mio. kroner.

12.2 Publication plan

The design of - and results from - the project will be published in scientific articles. In addition to original scientific papers, publication and presentation in non-academic settings will be prioritized, since the topic of this project is highly relevant to professionals within public health and antenatal care, and well-founded information is being urgently requested from the clinical field and policy makers.

<u>Article overview</u>

Authorship is determined according to the International Committee of Medical Journals Editors (ICMJE) guidelines. A design article of the trial will be published. Results from the trial will be published regardless of whether findings are positive or negative with an expected number of 2-3 papers per year.

12.3 Spin-off projects

If adequate financing can be obtained we wish to conduct:

PhD project examining associations and intervention effects in regards to antenatal and post-natal depression and anxiety among men and women.

PhD project related to in-depth process evaluation of the intervention.

Center for Intervention Research

Future studies on intervention effects on SOC, as various intervention studies including one addressing parental stress suggest that interventions may be effective in increasing SOC (115-117).

Future studies on intervention effects on self-rated health and subjective health complaints, as perceived stress, negative coping expectations, and inappropriate coping responses affect physiological processes that may permanently lower the threshold for experiencing subjective health complaints (118, 119). Self-rated health is a valid measure (120-123) that predicts mortality (124), morbidity, and the use of health services (125).

Future studies including health visitor data on parent-child interaction, which will require further data-sampling.

Long-term follow-up studies using national registers.

12.4 Intellectual property rights

Belong to the Centre for Intervention Research and The National Board of Health

13. Supplements

Overview of intervention design, overview of project model

Center for Intervention Research

Intervention design

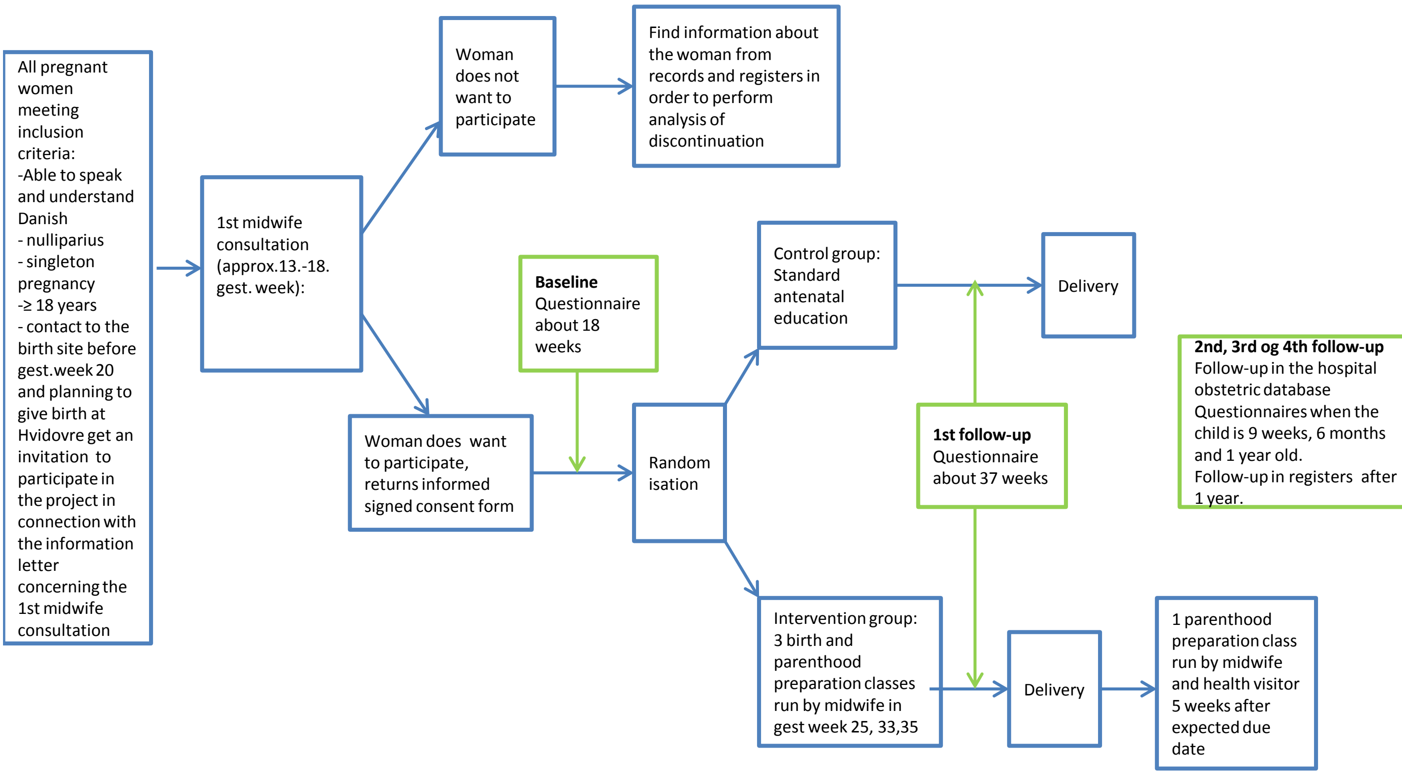

Center

Project model

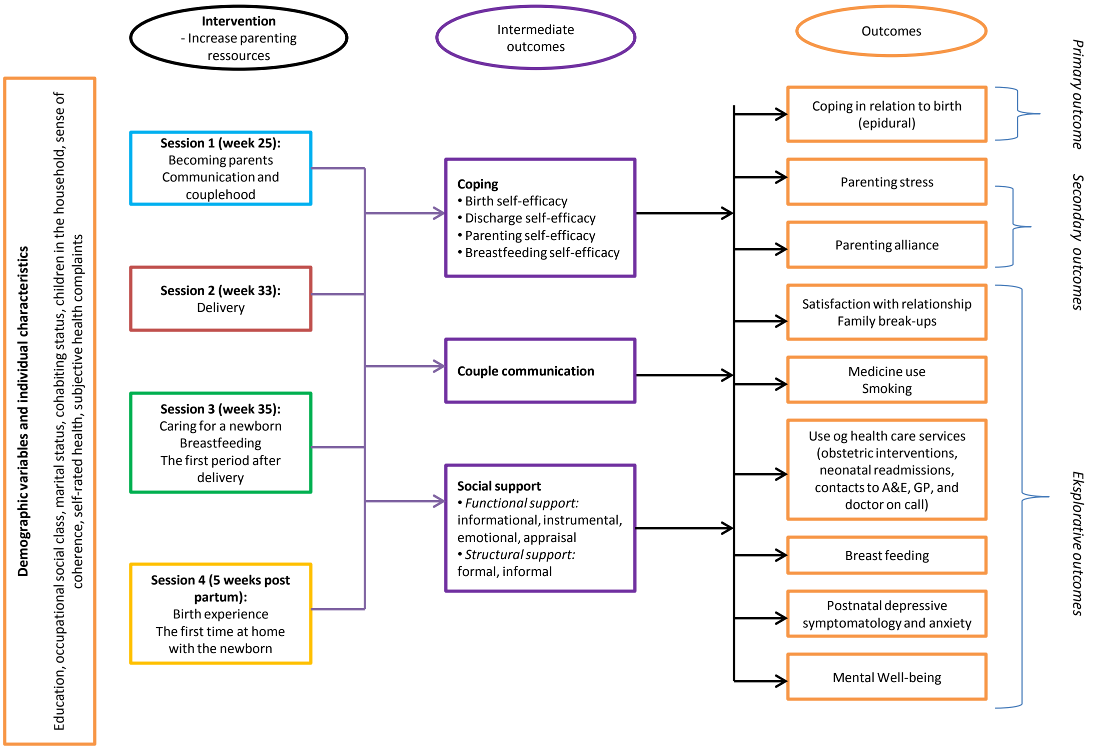

Center for Inter

14. References

Reference List

1. Cowan C, Cowan P. When partners become parents: The big life change for couples. Mahwah NJ: Lawrence Erlbaum Associates Publishers; 2000 2000.
2. Abidin R. The Determinants of Parenting Behavior. Journal of Clinical Child Psychology. 1992;21(4):407-12.
3. National Board of Health D. Status on regional and municipal offers in relation to pregnancy and delivery with focus on delivery and early discharge (In Danish: Status for regionernes og kommunernes tilbud til gravide og fødende med fokus på ambulante fødsler og tidlige udskrivelser). Copenhagen: 2011 2011. Report No.: j.nr. 7-205-03-31/1/chb.

4. Gagnon AJ, Sandall J. Individual or group antenatal education for childbirth or parenthood, or both. CochraneDatabaseSystRev. 2007(3):CD002869.
5. Barlow J, Parsons J. Group-based parent-training programmes for improving emotional and behavioural adjustment in 0-3 year old children. CochraneDatabaseSystRev. 2003(1):CD003680.
6. Assel MA, Landry SH, Swank PR, Steelman L, Miller-Loncar C, Smith KE. How do mothers' childrearing histories, stress and parenting affect children's behavioural outcomes? Child Care Health and Development. 2002;28(5):359-68.
7. Benzies KM, Harrison MJ, Magill-Evans J. Parenting stress, marital quality, and child behavior problems at age 7 years. Public Health Nursing. 2004;21(2):111-21.
8. Deater-Deckard K. Parenting stress and child adjustment: Some old hypotheses and new questions. Clinical Psychology-Science and Practice. 1998;5(3):314-32.
9. Nystrom K, Ohrling K. Parenthood experiences during the child's first year: literature review. JAdvNurs. 2004;46(3):319-30.
10. Schor EL. Family pediatrics: report of the Task Force on the Family. Pediatrics. 2003;111(6 Pt 2):1541-71.
11. Deater-Deckard K, Pinkerton R, Scarr S. Child care quality and children's behavioral adjustment: a four-year longitudinal study. JChild PsycholPsychiatry. 1996;37(8):937-48.
12. Dunkley J. Health promotion in midwifery practise: a resource for health professionals. Edinburgh: Bailliere Tindall; 2000 2000.
13. Biro MA. What has public health got to do with midwifery? Midwives' role in securing better health outcomes for mothers and babies. Women Birth. 2011;24(1):17-23.
14. Brot C, Poulsen A. Recommendations in relation to antenatal care 2009 (In Danish: Anbefalinger for svangreomsorgen 2009). Copenhagen: 2009 2009. Report No.
15. Haggman-Laitila A, Pietila AM. Perceived benefits on family health of small groups for families with children. Public Health Nurs. 2007;24(3):205-16.
16. Barlow J, Coren E. Parent-training programmes for improving maternal psychosocial health. CochraneDatabaseSystRev. 2004(1):CD002020.
17. Sanders MR. Triple P-Positive Parenting Program as a public health approach to strengthening parenting. JFamPsychol. 2008;22(4):506-17.
18. Lazarus R, Folkman S. Stress, Appraisal, and Coping. New York: Springer-Verlag; 1984 1984.
19. Belsky J. The determinants of parenting: a process model. Child Dev. 1984;55(1):83-96.

Center for Intervention Research

20. Bandura A. Self-efficacy: The exercise of control. New York1997 1997.
21. Coleman PK, Karraker KH. Parenting self-efficacy among mothers of school-age children: Conceptualization, measurement, and correlates. Family Relations. 2000;49(1):13-24.
22. Kendall S, Bloomfield L. Developing and validating a tool to measure parenting self-efficacy. Journal of Advanced Nursing. 2005;51(2):174-81.
23. Coleman PK, Karraker KH. Self-efficacy and parenting quality: Findings and future applications. Developmental Review. 1998;18(1):47-85.
24. Mash EJ, Johnston C. Determinants of Parenting Stress - Illustrations from Families of Hyperactive-Children and Families of Physically Abused-Children. Journal of Clinical Child Psychology. 1990;19(4):313-28.
25. Coleman PK, Karraker KH. Maternal self-efficacy beliefs, competence in parenting, and toddlers' behavior and developmental status. Infant Mental Health Journal. 2003;24(2):126-48.
26. Kronborg H, Vaeth M. The influence of psychosocial factors on the duration of breastfeeding. ScandJPublic Health. 2004;32(3):210-6.
27. Ystrom E, Niegel S, Klepp KI, Vollrath ME. The impact of maternal negative affectivity and general self-efficacy on breastfeeding: The Norwegian mother and child cohort study. Journal of Pediatrics. 2008;152(1):68-72.
28. Maimburg RD, Vaeth M, Durr J, Hvidman L, Olsen J. Randomised trial of structured antenatal training sessions to improve the birth process. BJOG. 2010;117(8):921-8.
29. Antonovsky A. Unravelling the mystery of health. San Francisco: Jossey-Bass; 1987 1987.
30. Antonovsky A. The structure and properties of the sense of coherence scale. SocSciMed. 1993;36(6):725-33.
31. Nutbeam D, Kickbusch I. Advancing health literacy: a global challenge for the 21st century. Health Promotion International. 2000;15(3):183-4.
32. Illeris K, Jarvis P, Kegan R, Engeström Y, Elkjaer B, Mezirow J, et al. Contemporary Theories of Learning: Learning Theorists...in their own words. New York: Routledge; 2009 2009.
33. Bartholomew LK, Parcel GS, Kok G, Gottlieb NH, Fernández ME. Planning Health Promotion Programs - An intervention mapping approach. 3 rd ed. San Francisco: Jossey-Bass; 2011 2011.
34. Whittakar K, Cowley S. An effective programme is not enough: a review of factors associated with poor attendance and engagement with parenting support programmes. Children and Society. 2010.
35. Milgrom J, Schembri C, Ericksen J, Ross J, Gemmill AW. Towards parenthood: an antenatal intervention to reduce depression, anxiety and parenting difficulties. JAffectDisord. 2011;130(3):385-94.
36. Freedman CM, Low SM, Markman HJ, Stanley SM. Equipping couples with the tools to cope with predictable and unpredictable crisis events: the PREP Program. International journal of emergency mental health. 2002;4(1):49-55.
37. Sundhedsoplysning Kf. Kort og godt om amning. 11 ed. Copenhagen, Denmark: Komiteen for Sundhedsoplysning 2013.
38. Wood L, Egger M, Gluud LL, Schulz KF, Juni P, Altman DG, et al. Empirical evidence of bias in treatment effect estimates in controlled trials with different interventions and outcomes: meta-epidemiological study. BMJ. 2008;336(7644):601-5.

Center for Intervention Research

39. Alehagen S, Wijma K, Wijma B. Fear during labor. Acta Obstetricia et Gynecologica Scandinavica. 2001;80(4):315-20.
40. Cohen S, Kamarck T, Mermelstein R. A global measure of perceived stress. JHealth SocBehav. 1983;24(4):385-96.
41. Monroe SM. Modern Approaches to Conceptualizing and Measuring Human Life Stress

2899. Annual Review of Clinical Psychology. 2008;4(1):33-52.
42. Cohen S, Tyrrell DA, Smith AP. Negative life events, perceived stress, negative affect, and susceptibility to the common cold

1. J PersSoc Psychol. 1993;64(1):131-40.
43. Monroe S, Kelley J. Measurement of stress appraisal. Measuring Stress: A Guide for Health and Social Sciences. New York: Oxford Univ. Press; 1995. p. 122-47.
44. Ostberg M, Hagekull B, Wettergren S. A measure of parental stress in mothers with small children: dimensionality, stability and validity. ScandJPsychol. 1997;38(3):199-208.
45. Ostberg M, Hagekull B. A structural modeling approach to the understanding of parenting stress. JClinChild Psychol. 2000;29(4):615-25.
46. McMahon C, Meins E. Mind-mindedness, parenting stress, and emotional availability in mothers of preschoolers. Early Childhood Research Quarterly. 2012;27:245-52.
47. Hadadian A, Merbler J. Mother's Stress: Implications for Attachment Relationships. Early Child Development and Care. 1996;125:59-66.
48. Jarvis P, Creasey G. Parental stress, coping, and attachment in families with an 18-month-old infant. Infant Behavior and Development. 1991;14:383-95.
49. Rodgers A. Multiple sources of stress and parenting behavior. Children and YouthServices Review. 1998;20:525-46.
50. Anthony LG, Anthony BJ, Glanville DN, Naiman DQ, Waanders C, Shaffer S. The relationships between parenting stress, parenting behaviour and preschoolers' social competence and behaviour problems in the classroom. Infant and Child Development. 2005;14(2):133-54.
51. Rodriguez C, Green A. Parenting stress and anger expression as predictors of child abuse potential. Child Abuse and Neglect. 1997;21:367-77.
52. Essex MJ, Klein MH, Cho E, Kalin NH. Maternal stress beginning in infancy may sensitize children to later stress exposure: effects on cortisol and behavior. BiolPsychiatry. 2002;52(8):776-84.
53. Milgrom J, McCloud P. Parenting stress and postnatal depression. StressMedicine. 1996;12:177-86.
54. Y L, S S, R K. The effect of parenting stress on marital quality. An integrated mother-father model. Journal of Family Issues. 1996;17:114-35.
55. Abidin RR, Brunner JF. Development of A Parenting Alliance Inventory. Journal of Clinical Child Psychology. 1995;24(1):31-40.
56. Nielsen NR, Kristensen TS, Schnohr P, Gronbaek M. Perceived stress and cause-specific mortality among men and women: results from a prospective cohort study. AmJEpidemiol. 2008;168(5):481-91.
57. Larsen F, al. e. Tidlig indsats mod fødselsdepression - erfaringer fra et projekt i Århus Kommune. Århus: 2009 2009. Report No.
58. Bennett HA, Einarson A, Taddio A, Koren G, Einarson TR. Prevalence of depression during pregnancy: systematic review. ObstetGynecol. 2004;103(4):698-709.

Center for Intervention Research

59. Gavin NI, Gaynes BN, Lohr KN, Meltzer-Brody S, Gartlehner G, Swinson T. Perinatal depression: a systematic review of prevalence and incidence. ObstetGynecol. 2005;106(5 Pt 1):1071-83.
60. Hickey D, Carr A, Dooley B, Guerin S, Butler E, Fitzpatrick L. Family and marital profiles of couples in which one partner has depression or anxiety. JMarital FamTher. 2005;31(2):171-82.
61. Storksen HT, Eberhard-Gran M, Garthus-Niegel S, Eskild A. Fear of childbirth; the relation to anxiety and depression. Acta ObstetGynecolScand. 2012;91(2):237-42.
62. Chung TK, Lau TK, Yip AS, Chiu HF, Lee DT. Antepartum depressive symptomatology is associated with adverse obstetric and neonatal outcomes. PsychosomMed. 2001;63(5):830-4.
63. Beck CT. The effects of postpartum depression on maternal-infant interaction: a meta-analysis. NursRes. 1995;44(5):298-304.
64. Avan B, Richter LM, Ramchandani PG, Norris SA, Stein A. Maternal postnatal depression and children's growth and behaviour during the early years of life: exploring the interaction between physical and mental health. ArchDisChild. 2010;95(9):690-5.
65. Goodman S, Brand S. Infants of depressed mothers, Vulnerabilities, Risk factors and Protective factors for the later Development of Psychopathology. In: CH Z, editor. Handbook of Infant Mental Health. 3rd ed. New York: The Guildford Press; 2011. p. 153-70.
66. Murray L, Sinclair D, Cooper P, Ducournau P, Turner P, Stein A. The socioemotional development of 5-year-old children of postnatally depressed mothers. JChild PsycholPsychiatry. 1999;40(8):1259-71.
67. Skovgaard A. Mental health problems and psychopathology in infancy and early childhood – An epidemiological study. Copenhagen: University of Copenhagen; 2010.
68. Beck CT. The effects of postpartum depression on child development: a meta-analysis. ArchPsychiatrNurs. 1998;12(1):12-20.
69. Stein A, Ramchandani P, Murray L. Impact of Parental Psychiatric Disorder and Physical illness. In: M R, editor. Rutter's Child and Adolescent Psychiatry: Blackwell Publishing; 2008. p. 407-20.
70. Elberling H. The Prevalence and predictors of mental health problems and disorders in a general population of 5-7 year old children. Copenhagen: University of Copenhagen; 2012.
71. Skovgaard AM, Olsen EM, Christiansen E, Houmann T, Landorph SL, Jorgensen T. Predictors (0-10 months) of psychopathology at age 11/2 years - a general population study in The Copenhagen Child Cohort CCC 2000. J Child Psychol Psychiatry. 2008;49(5):553-62.
72. Leahy-Warren P, McCarthy G, Corcoran P. Postnatal depression in first-time mothers: prevalence and relationships between functional and structural social support at 6 and 12 weeks postpartum. ArchPsychiatrNurs. 2011;25(3):174-84.
73. Leahy-Warren P. First-time mothers: social support and confidence in infant care. JAdvNurs. 2005;50(5):479-88.
74. Christensen A, Brixval C, Svendsen M, Laursen B, Holstein B. Breastfeeding in 14 municipalities - what factors are important for exclusive breastfeeding when the child reaches four months? (In Danish: Amning i 14 kommuner - Hvilke faktorer har betydning for fuld amning, når barnet er fire måneder?). Copenhagen: 2011 2011. Report No.
75. Holmes P, Oppenheimer LW, Wu Wen S. The relationship between cervical dilatation at initial presentation in labour and subsequent intervention. BJOG: An International Journal of Obstetrics & Gynaecology. 2001;108(11):1120-4.

Center for Intervention Research

76. Janicke DM, Finney JW. Children's primary health care services: A social-cognitive model of sustained high use. Clinical Psychology-Science and Practice. 2001;8(2):228-41.
77. Golding J, Pembrey M, Jones R. ALSPAC--the Avon Longitudinal Study of Parents and Children. I. Study methodology. PaediatrPerinatEpidemiol. 2001;15(1):74-87.
78. Schulz MS, Cowan CP, Cowan PA. Promoting healthy beginnings: a randomized controlled trial of a preventive intervention to preserve marital quality during the transition to parenthood. JConsult ClinPsychol. 2006;74(1):20-31.
79. Hildingsson I, Tingvall M, Rubertsson C. Partner support in the childbearing period -- a follow up study. Women Birth. 2008;21(4):141-8.
80. Ekholm O, Hesse U, Davidsen M, Kjoller M. The study design and characteristics of the Danish national health interview surveys. ScandJPublic Health. 2009;37(7):758-65.
81. Koushede V, Holstein BE, Andersen A, Holme HE. Stress and medicine use for headache: does sense of coherence modify the association? EurJPublic Health. 2010.
82. Koushede V, Ekholm O, Holstein BE, Andersen A, Hansen EH. Stress and use of over-the-counter analgesics: prevalence and association among Danish 25 to 44-year-olds from 1994 to 2005. IntJPublic Health. 2011;56(1):81-7.
83. Kristensen T. Facts and myths about stress (in Danish: Fakta og Myter om stress). National Research Centre for the Working Environment; 2007.
84. Abbott FV, Fraser MI. Use and abuse of over-the-counter analgesic agents. JPsychiatry Neurosci. 1998;23(1):13-34.
85. Johansen A, Jespersen L, Davidsen M, Michelsen S, Morgan C, Helweg-Larsen K, et al. Danish children's health and morbidity (In Danish: Danske børns sundhed og sygelighed). Copenhagen: 2009 2009. Report No.
86. Allotey P, Reidpath DD, Elisha D. "Social medication" and the control of children: a qualitative study of over-the-counter medication among Australian children. Pediatrics. 2004;114(3):e378-e83.
87. Tones K. Evaluating health promotion - Beyond the RCT In: Best Practices Quality and Effectiveness of Health Promotion. Tallinn: 2000 2000. Report No.
88. Raikes HA, Thompson RA. Efficacy and social support as predictors of parenting stress among families in poverty. Infant Mental Health Journal. 2005;26(3):177-90.
89. Weiss M, Ryan P, Lokken L. Validity and reliability of the perceived readiness for disharge after birth scale. JOGNN Clinical research. 2005;35:34-45.
90. Salonen AH, Kaunonen M, Astedt-Kurki P, Jarvenpaa AL, Isoaho H, Tarkka MT. Parenting self-efficacy after childbirth. JAdvNurs. 2009;65(11):2324-36.
91. Elek SM, Hudson DB, Bouffard C. Marital and parenting satisfaction and infant care self-efficacy during the transition to parenthood: the effect of infant sex. Issues ComprPediatrNurs. 2003;26(1):45-57.
92. Gross D, Tucker S. Parenting confidence during toddlerhood. A comparison of mothers and fathers. Nurse Pract. 1994;19(10):25, 9-4.
93. Jones TL, Prinz RJ. Potential roles of parental self-efficacy in parent and child adjustment: a review. ClinPsycholRev. 2005;25(3):341-63.
94. Teti DM, Gelfand DM. Behavioral competence among mothers of infants in the first year: the mediational role of maternal self-efficacy. Child Dev. 1991;62(5):918-29.
95. Ferketich SL, Mercer RT. Predictors of role competence for experienced and inexperienced fathers. NursRes. 1995;44(2):89-95.
96. Mercer RT, Ferketich SL. Experienced and inexperienced mothers' maternal competence during infancy. ResNursHealth. 1995;18(4):333-43.

Center for Intervention Research

97. Tarkka MT, Paunonen M, Laippala P. How first-time mothers cope with child care while still in the maternity ward. IntJNursPract. 2000;6(2):97-104.
98. Bloomfield L, Kendall S. Testing a parenting programme evaluation tool as a pre- and post-course measure of parenting self-efficacy. Journal of Advanced Nursing. 2007;60(5):487-93.
99. Bloomfield L, Kendall S. Audit as evidence: the effectiveness of '123 magic' programmes. Community Pract. 2010;83(1):26-9.
100. Svensson J, Barclay L, Cooke M. Randomised-controlled trial of two antenatal education programmes. Midwifery. 2009;25(2):114-25.
101. Caldera D, Burrell L, Rodriguez K, Crowne SS, Rohde C, Duggan A. Impact of a statewide home visiting program on parenting and on child health and development. Child Abuse Negl. 2007;31(8):829-52.
102. Salonen AH, Kaunonen M, Astedt-Kurki P, Jarvenpaa AL, Isoaho H, Tarkka MT. Effectiveness of an internet-based intervention enhancing Finnish parents' parenting satisfaction and parenting self-efficacy during the postpartum period. Midwifery. 2010.
103. Leahy-Warren P, McCarthy G. Maternal parental self-efficacy in the postpartum period. Midwifery. 2010.
104. Leahy-Warren P, McCarthy G, Corcoran P. First-time mothers: social support, maternal parental self-efficacy and postnatal depression. JClinNurs. 2011.
105. Due P, Holstein B, Lund R, Modvig J, Avlund K. Social relations: network, support and relational strain. SocSciMed. 1999;48(5):661-73.
106. Logsdon MC, Davis DW. Social and professional support for pregnant and parenting women. MCN AmJMaternChild Nurs. 2003;28(6):371-6.
107. Wilkins C. A qualitative study exploring the support needs of first-time mothers on their journey towards intuitive parenting. Midwifery. 2006;22(2):169-80.
108. Leahy-Warren P, McCarthy G. Postnatal depression: prevalence, mothers' perspectives, and treatments. ArchPsychiatrNurs. 2007;21(2):91-100.
109. Hildingsson IM. New parents' experiences of postnatal care in Sweden. Women Birth. 2007;20(3):105-13.
110. Reece SM. Social support and the early maternal experience of primiparas over 35. MaternChild NursJ. 1993;21(3):91-8.
111. Setterlind S, Larson G. The Stress Profile - A Psychosocial Approach to Measuring Stress. Stress Medicine. 1995;11(2):85-92.
112. Habroe M, Schmidt L, Holstein BE. Does childbirth after fertility treatment influence sense of coherence? A longitudinal study of 1,934 men and women. Acta ObstetGynecolScand. 2007:1-7.
113. Hospital H. Data from the obstetric database Hvidovre Hospital. 2012.
114. Nielsen L, Curtis T, Kristensen TS, Rod NN. What characterizes persons with high levels of perceived stress in Denmark? A national representative study. ScandJ Public Health. 2008;36(4):369-79.
115. Langeland E, Wahl AK, Kristoffersen K, Hanestad BR. Promoting coping: salutogenesis among people with mental health problems. Issues MentHealth Nurs. 2007;28(3):275-95.
116. Stokes RH, Holsti L. Paediatric occupational therapy: addressing parental stress with the sense of coherence. CanJOccupTher. 2010;77(1):30-7.

Center for Intervention Research

117. Vastamaki J, Moser K, Paul KI. How stable is sense of coherence? Changes following an intervention for unemployed individuals. Scandinavian Journal of Psychology. 2009;50(2):161-71.
118. Larsson G, Kallenberg KO. Sense of coherence socioeconomic conditions and health- interrelationships in a nation-wide Swedish sample. European Journal of public health. 1996;6:175-80.
119. Ursin H. Sensitization, somatization, and subjective health complaints. IntJBehavMed. 1997;4(2):105-16.
120. Chandola T, Jenkinson C. Validating self-rated health in different ethnic groups. EthnHealth. 2000;5(2):151-9.
121. Haddock CK, Poston WS, Pyle SA, Klesges RC, Vander Weg MW, Peterson A, et al. The validity of self-rated health as a measure of health status among young military personnel: evidence from a cross-sectional survey. Health QualLife Outcomes. 2006;4:57.
122. Rohrer JE, Herman DC, Merry SP, Naessens JM, Houston MS. Validity of overall self-rated health as an outcome measure in small samples: a pilot study involving a case series. JEvalClinPract. 2009;15(2):366-9.
123. Lundberg O, Manderbacka K. Assessing reliability of a measure of self-rated health. ScandJSocMed. 1996;24(3):218-24.
124. Idler EL, Benyamini Y. Self-rated health and mortality: a review of twenty-seven community studies. JHealth SocBehav. 1997;38(1):21-37.
125. Miilunpalo S, Vuori I, Oja P, Pasanen M, Urponen H. Self-rated health status as a health measure: the predictive value of self-reported health status on the use of physician services and on mortality in the working-age population. JClinEpidemiol. 1997;50(5):517-28.

Center for Intervention Research
